# Reinforcing the idea of an early dispersal of *Hippopotamus amphibius* in Europe: Restoration and multidisciplinary study of the skull from the Middle Pleistocene of Cava Montanari (Rome, central Italy)

**DOI:** 10.1371/journal.pone.0293405

**Published:** 2023-11-22

**Authors:** Beniamino Mecozzi, Alessio Iannucci, Marco Mancini, Daniel Tentori, Chiara Cavasinni, Jacopo Conti, Mattia Yuri Messina, Alex Sarra, Raffaele Sardella

**Affiliations:** 1 Dipartimento di Scienze della Terra (PaleoFactory lab.), Sapienza Università di Roma, Roma, Italy; 2 Department of Geosciences, Section of Terrestrial Palaeoclimatology, Eberhard-Karls-University Tübingen, Tübingen, Germany; 3 Istituto Di Geologia Ambientale E Geoingegneria, Consiglio Nazionale Delle Ricerche (CNR), Area della Ricerca di Roma 1, Monterotondo, Rome, Italy; 4 Dipartimento di Biologia Ambientale, Sapienza Università di Roma, Roma, Italy; 5 Polo Museale, Sapienza Università di Roma, Roma, Italy; 6 Cooperativa Fabrica Conservazione e Restauro, Roma, Italia; Università degli Studi di Torino, ITALY

## Abstract

A skull of *Hippopotamus* recovered from the area of Tor di Quinto, within the urban area of Rome (central Italy) is here redescribed. Despite being one of the most complete specimens of hippopotamuses of the European Pleistocene, the Tor di Quinto skull did not attract much research interest, due to long-standing uncertainties on its provenance. This work begun in 2021, when the skull was restored, within a large renovation project on the vertebrate exposed at the Earth Science University Museum of Sapienza University of Rome. Original sediments were found inside the cranial and mandible cavities during the restoration work, which were sampled for petrographic analyses. By combining a review of the old paleontological, archeological and geological literature published during the 19^th^ and 20^th^ century on the Rome basin and the correlation of these new sedimentological and petrographic information with the lithostratigraphic and synthemic units of the national geological cartography, we clarify that the *Hippopotamus* skull was most likely to have been collected from a quarry called Cava Montanari, from a formation dated between 560 and 460 ka. Morphological and biometric analyses clearly support an attribution of the Cava Montanari specimen to the extant species *Hippopotamus amphibius*. The reassessment of the stratigraphic and geological data on Cava Montanari implies that the studied specimen is the earliest confirmed occurrence of *Hippopotamus amphibius* in the European fossil record.

## Introduction

The hippopotamuses were widely diffused in Europe during the Quaternary, with an earliest dispersal recognized during the middle Villafranchian around 2.2 Ma (“Hippo event” *sensu* [1]). Hippopotamuses are iconic animals, often taken as representatives, together with elephants, of the savannah grasslands ecosystem, nowadays typical of the African continent. The presence of hippopotamuses is often related with warm climatic conditions, but this relationship does not express completely their ecological profile. Modern hippopotamuses are especially dependent on the presence of water, and hence indicators of humid conditions and mild winters [2–7]. Therefore, findings of hippopotamuses in Quaternary deposits indicate the presence of water, in the form of lakes, ponds or rivers. Despite the long persistence of hippopotamuses in Europe, their remains are heterogeneously distributed in the fossil record, with a few nearly complete skeletons found in lacustrine or, rarely, fluvial deposits [e.g., 5, 8, 9] testifying to their unique bond with water bodies. The ancestor of the extant *Hippopotamus amphibius*, *Hippopotamus antiquus*, displayed morphological features interpreted as indicative of a pronounced adaptation to an aquatic lifestyle, as confirmed by dietary proxies [10–12].

Decades of debate interested the taxonomy of European Pleistocene hippopotamuses and different scenarios on the evolution of the genus *Hippopotamus*. Several species have been established, islands excluded, among which: i) *Hippopotamus major*, proposed by Cuvier (1824) to identify the large Early and early Middle Pleistocene forms; ii) *Hippopotamus tiberinus*, introduced by Mazza (1991) to highlight the affinity of some Middle Pleistocene specimens with the extinct African Early Pleistocene species *Hippopotamus gorgops*; iii) *Hippopotamus incognitus*, erected by Faure (1984) to identify the small Middle and Late Pleistocene forms [7, 13–17]. In general, these forms are no longer considered valid species, but often synonyms of other taxa. The schematic model proposed by [17], adopted in many works (e.g., [7, 18–23], but see [24]), involves only two species: *H*. *antiquus* (= *H*. *major* = *H*. *amphibius antiquus = H*. *tiberinus = H*. ex gr. *antiquus = H*. *georgicus*); and *H*. *amphibius (= H*. *incognitus = H*. *amphibius incognitus)*.

**Fig 1:**
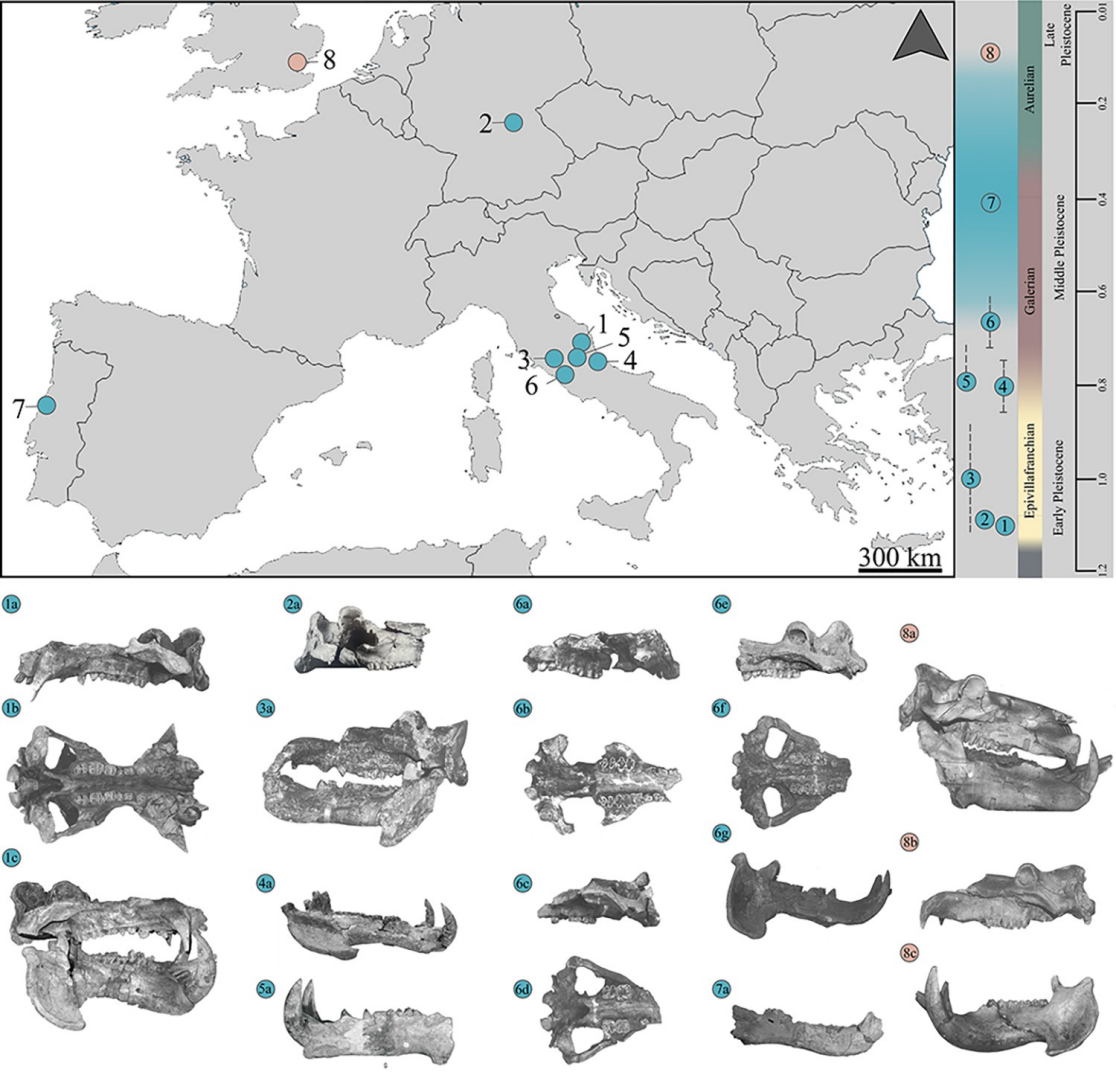
Selected cranial and mandible remains of late Early to Late Pleistocene hippopotamuses from Europe. 1–108, skull from Collecurti in left lateral (a), ventral (b) and right lateral (c) views; 2—IQW 1991/23 (Mei 23 438), cranium of Untermassfeld in right lateral view (a); 3 –MPUR/V 1950, skull from Sant’Oreste in left lateral view (a); 4 –No catalogue number, mandible from Ortona in right lateral view (a); 5—MPUR/V 52, mandible from Vallinfreda in left lateral view (a); 6 –Crania from La Maglianella; 321, cranium in left lateral (a) and ventral (b) views; 322, cranium in right lateral (c) and occlusal (d) views; 601, skull in left lateral (e), occlusal (f) and right lateral (g) views; 7 –MG3665, hemimandible from Condeixa in right lateral view (a); 8 –Crania and mandibles from Barrington; D3980, skull, D13938 in right lateral view (a); D13938, cranium in left lateral view (b); D3975, mandible right lateral view (c). Images modified from [8, 9, 15, 16, 21, 124, 125]. Images are not in scale. Colors: light blue—*Hippopotamus antiquus*; light red—*Hippopotamus amphibius*.

The *H*. *antiquus-H*. *amphibius* transition occurred in Europe during the Middle Pleistocene (ca. 600–128 ka), but this event is still vaguely understood. [4] and [25] already discussed the importance of identifying the earliest dispersal of *H*. *amphibius* in Europe, but after forty years this issue is still unsolved. The main obstacle concerns the criteria for the specific identification of fossil hippopotamuses, being the main diagnostic morphological features mainly observable in the skull. As reported in Fig 1, only a few cranial and mandibular remains have been described from fossil deposits dated to the second part of the Middle Pleistocene. During this time span, the majority of hippopotamuses fossil remains was found associated with lithic artefacts attributed to Early Paleolithic (see [21] for discussion). This is not a secondary aspect, since human exploitation often result in a selective accumulation of fossil remains. By combining the fragmentary nature of available fossil specimens and the lack of cranial material, these Middle Pleistocene records are considered of poor taxonomic value [21].

Considering reliable cranial and mandibular fossils, the last occurrence of *H*. *antiquus* is from La Maglianella (Rome, central Italy; [15, 16, 26]), dated approximately at 600 ka [27]. Recently, a mandible from the Middle Pleistocene site of Condeixa (Portugal, ca. 400 ka) was attributed to *H*. *antiquus* [21], which would seem to indicate a later persistence of this species in Iberian Peninsula.

Moving back to the early dispersal of *H*. *amphibius* in Europe, two main different hypotheses were proposed, and both resolve around the skull from Tor di Quinto (Rome, Italy). This specimen was firstly figured by [28] and attributed to Cava Montanari (“cava” meaning quarry in Italian). Later, [8] confirmed the attribution of the Tor di Quinto skull to *H*. *amphibius*, but they doubted its provenance from Cava Montanari, being unaware of the existence of a quarry in that area. The authors considered likely that the skull was collected from another quarry, Cava Nera Molinario, known to have yielded other mammal remains, attributed to *Cervus elaphus* and *Palaeoloxodon antiquus*, and dated to the Middle Pleistocene (between 530 and 500 ka approximately, MIS 13, [27]). Following this, several authors placed the oldest diffusion of *H*. *amphibius* within MIS 13 (e.g., [8, 17, 29]). Considering its unclear geographical provenance, [16] excluded the Tor di Quinto skull from the reconstruction of the evolution of the genus *Hippopotamus*, identifying the sample from the early Late Pleistocene of Barrington (MIS 5; United Kingdom) as the earliest occurrence of *H*. *amphibius* in Europe.

In this paper, we revise the skull from the Tor di Quinto area (Rome, central Italy) and we clarify its geographical provenance. This project begun in 2021, within a large restoration and renovation work of the fossil vertebrates stored at the Earth Science University Museum (MUST) of Sapienza University of Rome [30]. During the restoration activity, original sediment was found in cranial and mandible cavities, around the upper and lower teeth and on the outer side of the left hemimandible. The aims of our paper are: i) to review the old paleontological, archeological and geological literature published during the 19^th^ and 20^th^ century on the Rome basin to better define the geographical provenance of the *Hippopotamus* skull from Tor di Quinto; ii) to describe the sediment found during the restoration work and to perform petrographic analyses in order to add information about the original deposit from which this fossil was collected; iii) to provide chronological constrains through the correlation of these new sedimentological and petrographic information with the lithostratigraphic and synthemic units of the national geological cartography (CARG Project; [35]); iv) to carry out new morphological and biometric analysis to provide an update taxonomic appraisal of the specimen.

### Cava Montanari and its long history

The Tor di Quinto area is located on the right bank of the Tiber River, in the northern periphery of Rome. Nowadays, the original landscape of this territory is illegible, due to the intense urbanization. The Tor di Quinto area was investigated since the second half of the 19^th^ century, when a number of quarries were opened for the extraction of gravel as building material. [31] firstly reported the presence of mammal fossil remains in the same gravel levels. A pioneering work was published by [32], who illustrated and correlated several sedimentary deposits of the city of Rome, including the Tor di Quinto area, carrying out the first synthesis of the geological evolution of the Rome basin.

The main paleontological and archeological deposits described in the old literature are Ponte Molle, Tor di Quinto (= Torretta di Quinto), Cava Bertazzi, Cava del Selcio and Acquatraversa (e.g., [31–33]). Another quarry was described by [26], Cava Nera Molinario, located along the Flamina road. The last deposit in this area is Via Flamina km 8.2, described by [34].

Fossil remains from the Tor di Quinto area were generally referred to Middle Pleistocene and in particular to the Valle Giulia Formation (*sensu* [35]): Cava Nera Molinario (ca. 500 ka, MIS 13; [27]); Tor di Quinto (between 560–500 ka, MIS 14–13; [36]); Via Flaminia km. 8.2 (ca. 533 ka, MIS 13; [37]); Ponte Molle (between 540–460 ka; MIS 13, [33]).

The geographical provenance of the hippopotamus skull of Tor di Quinto was debated, and several authors preferred to exclude this specimen for the reconstruction of the evolution of the genus *Hippopotamus*. This skull was firstly figured by [28], when the authors carried out an overview on the history of the Geological and Paleontological Museums of Sapienza and their collections. The caption of their Fig 12 (pag. 26) said: “Cranio, visto di fronte e di lato, di *Hippopotamus amphibius*. Cava Montanari; Tor di Quinto (Roma) (circa 1/10 della grandezza naturale)” [Skull, in frontal and lateral views, of *Hippopotamus amphibius*.*–*Cava Montanari; Tor di Quinto (Roma) (ca. 1/10 of the life size)”].

[8] carried out the first description of this skull, confirming its attribution to *Hippopotamus amphibius*. In the footnote of pag. 91, the authors wrote: “In Fabiani & Maxia (1953) questo cranio e la mandibola sono indicati come provenienti da Cava Montanari, Tor di Quinto, mentre il cartellino associati ai reperti porta la dicitura “Ghiaie di Cava Nera Molinario, Tor di Quinto, Roma”. Agli scriventi non è risultata l’esistenza di una “Cava Montanari” nella zona di Tor di Quinto. Si ritiene pertanto anche, se in forma dubitativa, che questi resti possano provenire dai livelli ghiaiosi di Cava Nera Molinario” [In Fabiani & Maxia (1953) this cranium and mandible are referred to Cava Montanari, Tor di Quinto, while the label reports “gravels of Cava Nera Molinario, Tor di Quinto, Roma”. The authors do not know “Cava Montanari” in the Tor di Quinto area. Therefore, it is considered, even if with doubts, that these remains can be attributed to the gravel deposits of Cava Nera Molinario].

Nevertheless, it is worth mentioning that the same authors referred this skull to Cava Montanari in the main text (pag. 112, in [8]).

In 1995, Mazza published a large revision of European hippopotamuses, and stated that “the chronological attribution of the Tor di Quinto specimen is unreliable, because based on a too doubtful stratigraphic record”. After this work, the unclear geographical provenance divided the authors, and most of them ruled out this record for biochronological purposes.

In order to better define the geographical provenance of the skull from Tor di Quinto, we review the old paleontological, archeological and geological literature of the Rome basin published between the end of 19^th^ and the first half of the 20^th^ century. First, however, we have to say that the original label associated with the skull was lost, and the catalogue of the Earth Science University Museum, Sapienza University of Rome (including the former Paleontological Museum) has no information about the year of acquisition and the toponym.

The knowledge of hippopotamuses findings of the Rome basin was synthetized by [38]. The author cited 59 localities where hippopotamuses remains were collected, but no fossil was reported from Cava Montanari or Cava Nera Molinario [38]. The author mentioned other fossils from the Tor di Quinto area, recovered at Ponte Molle, or near this district, namely from Monte Antenne or Acquatraversa.

Following this, we could hypothesize that the skull from Tor di Quinto was discovered and become part of the collection of the Paleontological Museum of Sapienza after the work of [38] and before the work of [28].

**Fig 2:**
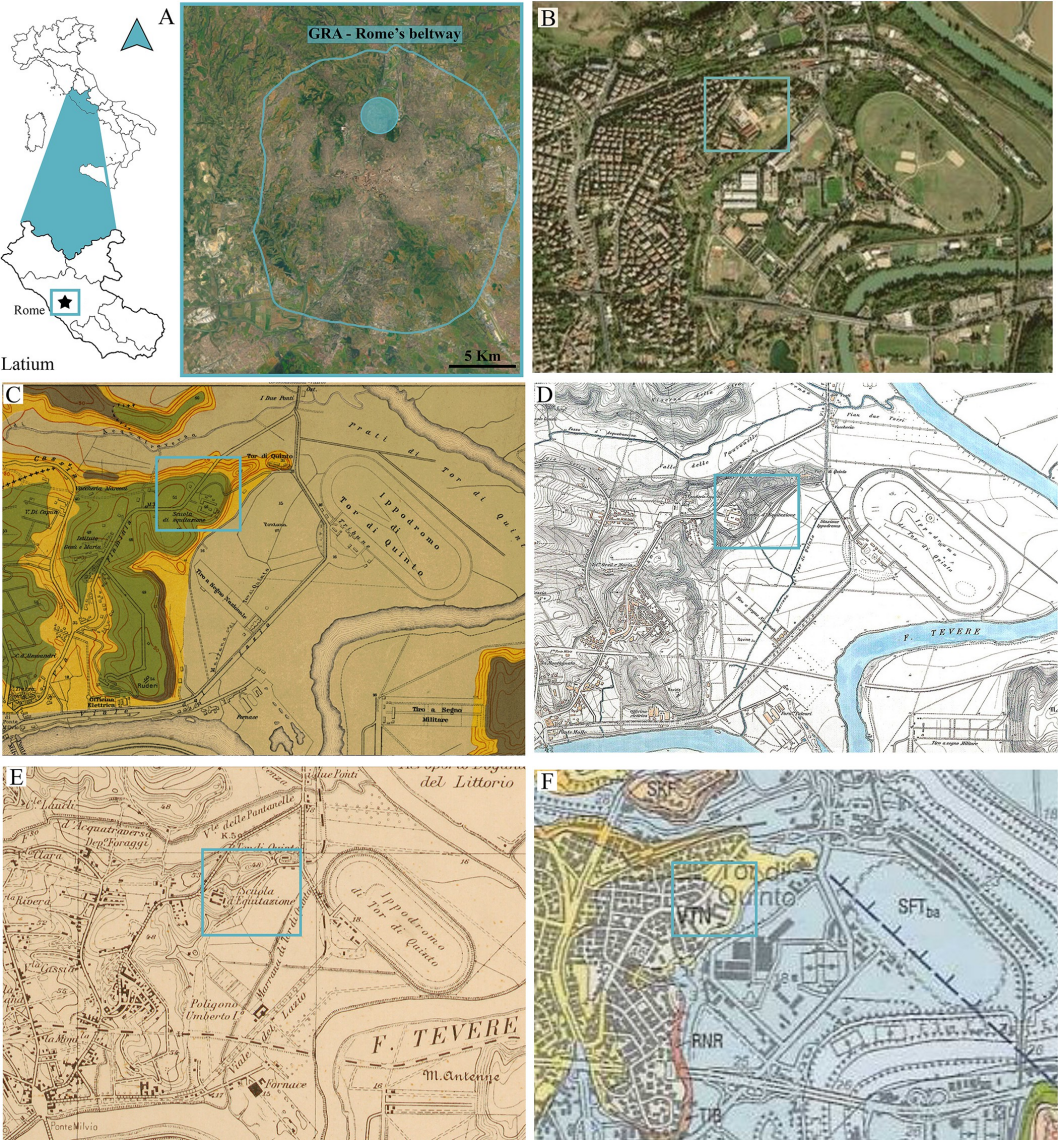
Location of the studied area of Tor di Quinto (A [modified by 100]-B [taken by USGS National MAP Viewer]. Sketches of the historical geological and topographic maps by [126] (C), [127] (D), [128] (E), and [35] (F). In the blue bounded square it is reported the location of the former military riding school (“Scuola di Equitazione”).

Of extraordinary importance for the aim of our work is the paper published by Rellini in 1932. The author reported the presence of a lithic tool and a tusk of *Elephas* (= *Palaeoloxodon*) *antiquus* at Cava Montanari, a quarry located near the “Scuola di Equitazione” (riding school), in the Tor di Quinto area [39]. This paper confirmed the presence of a quarry in the Tor di Quinto area called Cava Montanari, and the presence, albeit sporadic, of archeological and paleontological material. The Scuola di Equitazione is a military building, represented in the historical geological and topographic maps, and still present along the Flaminia road (nowadays called “Caserma Cap. Camillo Sabatini; Reggimento Lancieri di Montebello 8°”; Fig 2).

**Fig 3:**
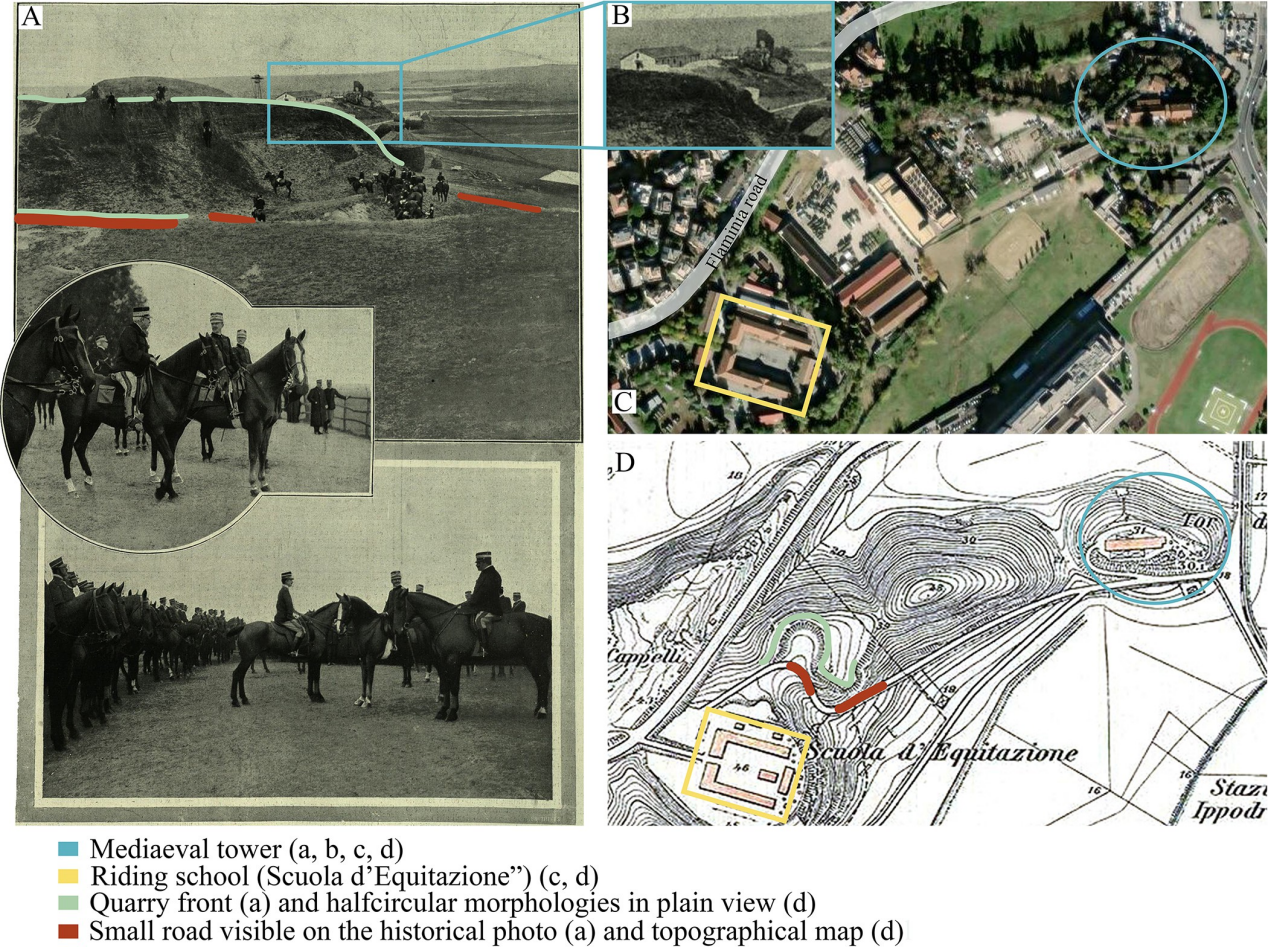
Historical picture of the Tor di Quinto area from the archive of the Istituto Superiore per la Protezione e la Ricerca Ambientale (ISPRA, A, B) and the same area today (taken by USGS National MAP Viewer, C) and from topographic maps by [127] (D).

Surveying the archival documentation, we found a historical picture in the data bank of the Istituto Superiore per la Protezione e la Ricerca Ambientale (ISPRA, Institute for Environmental Protection and Research), published in an Italian daily magazine named “L’Ilustrazione Italiana” [40]. The picture shows the then King of Italy, Vittorio Emanuele III, who went to ride a horse in the proximity of the riding school near the Flamina road, as reported in the caption (Fig 3). On the background of the photo, above on the left (Fig 3A), an old tower (named Tor di Quinto) can be observed, which is still present today and located at the crossroad between two streets, Via Tor di Quinto and Via del Casale di Tor di Quinto (light blue circle in Fig 3C). Considering this, we can infer that the picture was taken from the riding school toward the old tower to the North-Northeast. Therefore, the scarps on the hillslope, observable in Fig 3A, and with natural sediments exposed, probably corresponds to the quarry front of Cava Montanari, located close the riding school [39]. Examining the photograph, horizontal-bedded granular sediments (i,e, sands and pebbles) can be distinguished from the grass.

When we carried out the synoptic observation of old maps, from the oldest to the youngest, was possible to detect changes in the shape of the topographic contour lines (i.e., from a convex to a concave one). To better identify the geographical position of Montanari quarry, we apply the method proposed by [41], where the halfcircular morphologies in plain view are interpreted as ancient quarry fronts and floors.

Collectively, considering the historical map (Figs 2 and 3), the old picture (Fig 3A) and the information reported by [39], we know that Cava Montanari was in the proximity of the riding school, near a small road. As we can see in Fig 2D, 2E and 3D, a halfcircular morphology is present in front the riding school, separated by secondary road. This point should correspond to Cava Montanari.

### Geological setting

The study area of Tor di Quinto, in the northern periphery of Rome, is a terraced hilly relief located on the west bank of the Tiber River, within the Roman Basin. The Roman Basin is of tectono-sedimentary origin, straddles some 100 km in the NW-SE direction and is bordered by NNW-SSE and NE-SW trending normal faults. It is a hinterland basin located in the convergent belt between the Eurasian Plate and the subducting Adria Microplate. It was developed in the Early Pliocene-Holocene after the crustal extension affecting the western Northern-Central Apennines since Late Miocene, in connection with the progressive opening of the Tyrrhenian Sea backarc Basin and of the Adria slab retreat and roll-back [42–44].

In the Roman Basin the infill was mainly controlled by syn-rift subsidence in the Early Pliocene-Early Pleistocene (Zanclean pp.-Calabrian), and by the regional, post-rift, uplift of the western Apennine belt since the late Early Pleistocene (at about 1.1 Ma), thus recording the progressive transition from the open marine to the coastal and fluvial sedimentation.

The regional uplift, indeed, caused the emersion of the peri-Tyrrhenian belt and the development and integration of the ancient Tiber river’s drainage, which occurred in concomitance with the onset of the Sabatini Mts and Albani Hills volcanic activity of the Roman Magmatic Province (see [45], with references therein), respectively north and south of Rome.

The high frequency glacio-eustatic cycles of sea level oscillation and cycles of climate changes (80–100 ka spaced) exerted a complementary control on the syn-uplift and syn-volcanic fluvial and coastal sedimentation and erosion. This led to a terraced landscape bordering the Tiber Valley at the outer margin of the volcanic plateaux, and to a complex stratigraphic architecture with alternating incised-valley fills and interfluves [46–49], where fluvial sediments are preserved interbedded with distal pyroclastic deposits.

The Tor di Quinto terrace is composed by a set of purely sedimentary (i.e. active fluvial channels and floodplain facies) and pyroclastic lithostratigraphic units of Middle Pleistocene age, unconformably lying on the marine Pliocene-Early Pleistocene substratum: Fosso della Crescenza Fm, the *Tufo giallo della via Tiberina* (550 ka) Valle Giulia Fm, Palatino Unit (433 ka) *Tufo Rosso a scorie Nere sabatino* Vitina Formation [33, 35, 50].

#### Present day setting and modern sand detrital signature

**Fig 4:**
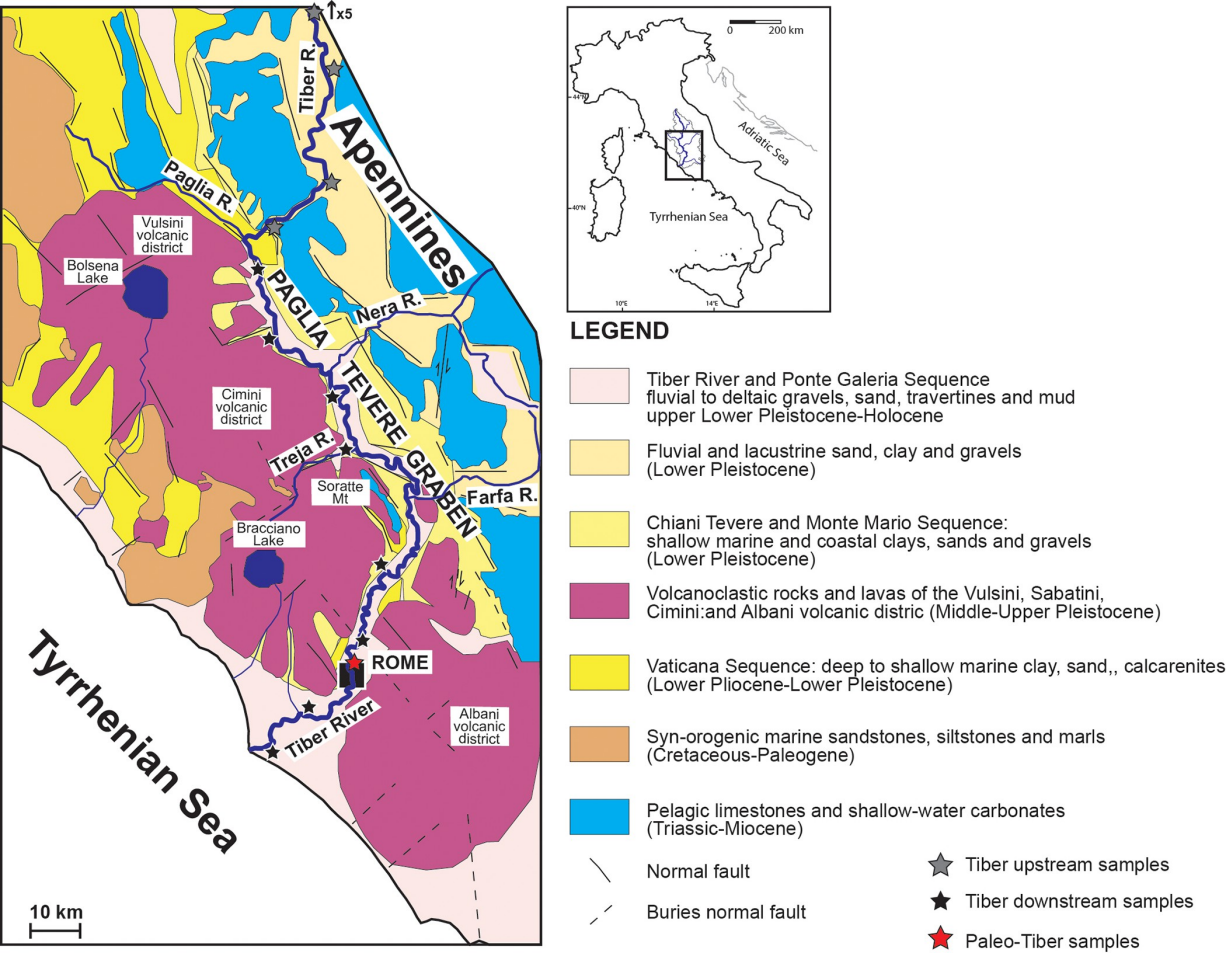
Geological sketch map of the middle and downstream Tiber River drainage basin (modified after [67]). Location of the upstream and downstream modern and ancient Tiber River samples used for petrographic analysis are reported.

At present, the Tiber River originates in the Apennine Mountains of Emilia Romagna and flows for 406 kilometers through Umbria and Lazio to the Tyrrhenian Sea (Fig 4). The modern Tiber River drainage basin extends for 17,375 km^2^ and comprises a large portion of the central Apennines in which carbonate, siliciclastic, and volcanic rocks, varying in age from Mesozoic to Quaternary, are present. The Apennine belt includes ophiolitic sequences and continental-margin successions consisting largely of Meso-Cenozoic pelagic to platform carbonate deposits associated with synorogenic turbiditic sediments deposited during the Oligocene–Miocene in foreland-basin systems. The sandy turbidite compositions range from feldspatho-quartzose to litho-feldspatho-quartzose with common metamorphic, plutonic, sedimentary, and volcanic lithic fragments [51, 52]. The middle and downstream catchment basin of the Tiber River and its tributaries lies in the magmatic Latium–Campania Superprovince [53], and the composition of Tiber River sand reflects the recycling of Miocene turbidite detritus, the widespread exposure of Mesozoic carbonates, and the more recent Quaternary potassic and ultrapotassic volcanism of the Roman magmatic province. Data from [53–56] indicate that the present Tiber River and delta contain: quartz, feldspars, and minor terrigenous lithic fragments from foredeep turbidites; limestone and chert from the Umbria pelagic succession; and subordinate volcanic detritus including volcanic lithic fragments, pyroxene grains, and leucite and sanidine crystals. These main components are those that, in different percentages, we have recognized in our analysis of the Roman basin alluvial deposits.

## Materials & methods

The hippopotamus skull MPUR/V 149 is housed in the University Museum of Earth Science, Department of Earth Sciences, Sapienza University of Rome (MUST, including the former Paleontological Museum, MPUR).

This skull was restored during the 2021, as part of the restoration work carried out on several mammal fossil skeletons exposed at the MUST [30]. During this activity, the integration applied in the past was removed as well as the colouring products that masked the original configuration of the skull (see S1 File). In addition, we found the original sediment that partially filled the cranial and mandible cavities or encrusted the basal part of upper and lower teeth and the outer side of the left hemimandible. These sediments, exactly those recovered from the inner part of the cranium and mandible cavities, were sampled and analyzed for the first time in this work.

### 3D Models

In order to obtain a virtual surface and highlight the morphology and the distribution of the clasts embedded on it, a 3D model of has been carried out by close-range photogrammetry.

Due the fragility and the dimension of the specimen, the photos were taken in the museum, without moving the specimen, directly on its base, using the “walk-around” technique described by [57].

65 photos were taken and imported in the Agisoft Metashape software at the Polo Museale Sapienza, in order to recreate a digital version of the surface, which initially consisted of a dense cloud of 13 million points.

The 3d shape of the mandible was created at high quality and consists in an open surface composed by more than 1.300.000 faces.

**Fig 5:**
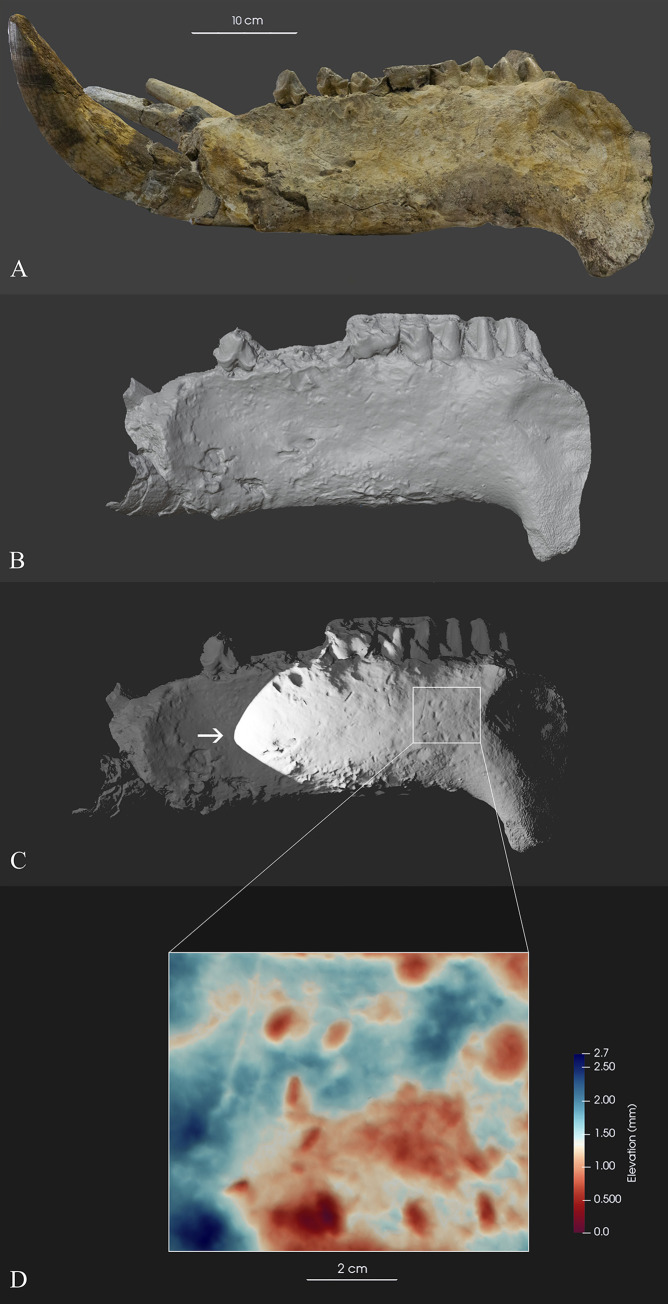
3D model of the left hemimandible of *Hippopotamus amphibius* from Cava Montanari (A-C) and the depth-map (D). Legend of the depth map: blue is the bone surface and the red pits are where pebbles had been pushed against and into the bone forming surface depressions.

The final 3D model was imported into Blender software where it was scaled and then exported as an obj file. In Blender, a light was set at an angle to the model in order to emphasize the surface details (Fig 5B and 5C). The obj file was then imported into the ParaView software, where a depth map of a portion of the outer surface of the jaw was made (Fig 5).

The further models of the entire cranium and the two hemimandibles have been carried out with the optical scanner Artec Eva (S1–S3 Appendices). The models, digitalized at MUST (University Earth Science Museum) of Sapienza, University of Rome, have been optimized for the publication and are composed by 900.000 faces. The original models are now stored into the digital collection of the museum.

### Petrographic analysis

**Fig 6:**
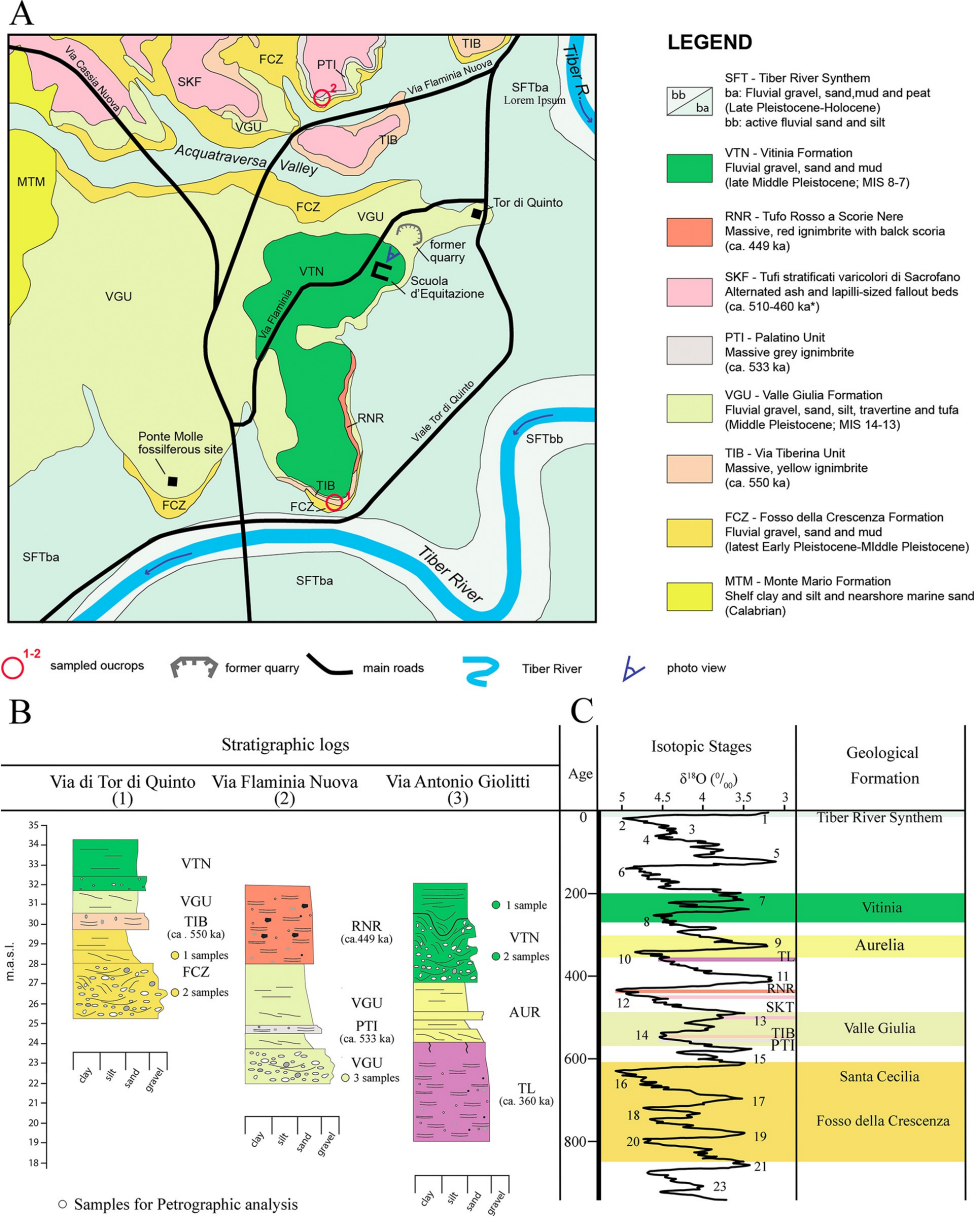
Geological map of the studied area (Tor di Quinto, Rome) after [35], with the location of the riding school (“Scuola d’Equitazione”), sampled outcrops, and the ancient quarry (A). Stratigraphic logs of the sample outcrops in this work (B). Chronostratigraphic scheme of the Geological Formation of the Rome basin (C).

Five samples were analyzed to characterize the sand composition of the sediment fraction recovered during restoration work on the surface of the *Hippopotamus* skull. The results were compared with newly acquired petrographic data from nine sediment samples collected from three different outcrops within the Roman basin (Fig 6). The outcrops were selected to characterize the sediment signature of the three Formations identified as the potential sedimentary units hosting the *Hippopotamus* skull. In particular, three samples per unit were collected from fluvial conglomerates, sand and silt deposits and fluvial-palustrine calcareous and volcanoclastic sands and silt levels belonging to the Fosso della Crescenza (FCZ), Valle Giulia (VGU) and Vitinia (VTN) Formations in the Tor di Quinto (North of Rome) and Mezzocamino (South of Rome) areas (Fig 6). In addition, detrital signatures were compared with previously published data from the Middle Pleistocene fluvial and nearshore sand and Holocene to Modern Tiber River sand [53–56].

**Fig 7:**
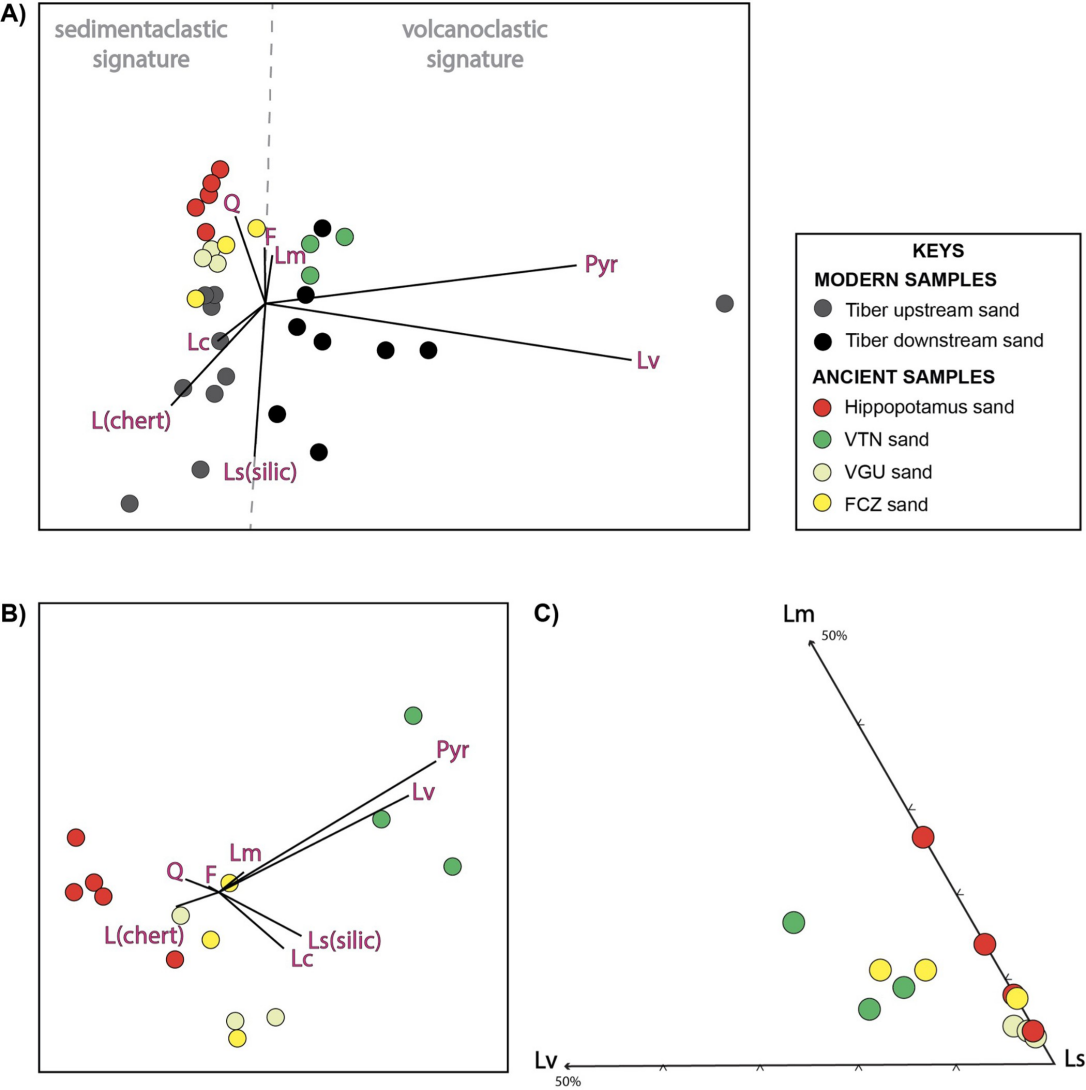
Compositional biplots [63] displaying data from modern Tiber upstream and downstream sand and ancient sand samples from the Vitinia (VTN), Valle Giulia (VGU), and Fosso della Crescenza (FCZ) Formations (A and B). Rays represent the parameters in the datasets (Q, total quartz; K, K-feldpsar; P, plag; Lc, carbonate lithics; Lm, metamorphic lithics; Lv, volcanics; Hm, dense minerals). The length of each ray is proportional to the variability of the parameter in the data set; the angle between two rays reveals whether the corresponding parameters are well correlated (0°), uncorrelated (90°), or inversely correlated (180°) (from [64]). Note in A) that the modern upstream samples cluster with the *Hippopotamus*, VGU, and FCZ samples; conversely volcanoclastic downstream fluvial samples cluster with VTN sand. LmLvLs ternary diagram comparing the ancient sand lithic fragments composition. Note that the hippopotamus sediment samples overlap the lithic signature of the VGU sand (C).

Sand samples were sieved with a broad sand-size-range (125–500 μm) to include a grain-size window representative of the studied deposits. Quantitative sand composition was defined using the Gazzi-Dickinson method [58] and counted grains grouped into monomineralic and polymineralic categories. Recalculated parameters were plotted on the ternary diagram of Fig 7A and 7B and compositional biplot of Fig 7C to investigate modal compositions and the relative percentages of the main grain types and lithic fragments. LmLvLs ternary plot depicts the relative proportion among the main lithic fragments types (Lm, metamorphic lithic fragments; Ls, sedimentary lithic fragments, Lv, volcanic lithic fragments). The compositional biplot display point count data collected on sand samples and the main compositional parameters (rays; Q, total quartz; K, K-feldpsar; P, plagiocase feldspar; Lc, carbonate lithics; Lm, metamorphic lithics; Ls(silic), siliciclastic lithics; Lv, volcanics; Pyr, pyroxene phenocrists phases). The length of each ray is proportional to the variability of the parameter in the data set; the angle between two rays reveals whether the corresponding parameters are well correlated (0°), uncorrelated (90°), or inversely correlated (180°) [59, 60].

### Paleontological analysis

Morphological and biometric comparisons of the Tor di Quinto skull with corresponding specimens of other fossils and extant *Hippopotamus* [8, 14, 16, 61] were carried out, to evaluate its taxonomic attribution. The dental terminology adopted in this work follows [16, 62].

Following [16] 24 cranial variables have been measured: prosthion-nuchal crest length (LPN), prosthion-occipital condyle length (LPOc), prosthion-basion length (LPB), canine alveolus-nuchal crest length (LCN), narial notch-orbital cavity length (LnnOr), canine alveolus-orbital cavity length (LCOr), zygomatic arch-orbit height (HZOr), opisthion-akrocranion height (HOpA), basion-akrocranion height (HBA), height of the foramen magnum (HFm), breadth of the foramen magnum (BFm), breadth of the nuchal crest (BN), otion-otion breadth (Botot), bredth across the occipital condyles (BOc), breadth between the temporal lines (BTl), frontal breadth (BF), zygomatic breadth (BZ), breadth between the orbital cavities (BOrOr), infraorbital breadth (BiOr), breadth between the canine alveoli (BCa), breadth between the second incisor alveoli (BI2a), breadth between the second premolar alveoli (BP2), breadth between the first molar alveoli (BM1), breadth between the third molar alveoli (BM3) (Table 1).

**Table 1:** Craniodental measurements of *Hippopotamus* skull from Cava Montanari (Rome, central Italy).

Cranial measurements		Upper teeth measurements			Lower teeth measurements		
			Sx	Dx		Sx	Dx
**LPN**	625.3	**I1L**	32.7	32.8		41.1	41.1
**Lpoc**	639.5	**I1B**	39.1	39.5		43	38
**LPB**	567.5	**I2L**	29.0	27.8		27.5	
**LCN**	566.3	**I2B**	38.1	30.4		26.5	
**LnnOr**	307.3	**CL**	38.0	38.5		61.1	61.0
**LCOr**	330.5	**CB**	46.1	46.0		35.5	36.8
**HZOr**	119.3	**OT**	236.9		**OT**	310.0	
**HOpA**	140.3	**OP**	101.4		**OP**	125.4	
**HBA**	176.8	**OM**	136.5	134.6	**OM**	188.8	
**HFm**	43.5	**P2OL**	35.3		**P2OL**		
**BFm**	63.0	**P2IL**	35.1		**P2IL**	41.8	
**BN**	167.2	**P2AB**	28.0		**P2AB**	23.5	
**Bbotot**	298.5	**P2PB**	22.6		**P2PB**	28.4	
**BOc**	132.4	**P3OL**	35.2	34.8	**P3OL**		44.8
**BTl**		**P3IL**	36.9	35.6	**P3IL**		43.0
**BF**	7.8	**P3AB**	26.2	27.5	**P3AB**		23.5
**BZ**	410.3	**P3PB**	28.5	29.6	**P3PB**		28.6
**BOr-Or**	204.6	**P4OL**	27.0		**P4OL**		43.8
**BiOr**	124.5	**P4IL**	28.2		**P4IL**		42.5
**BCa**	216.4	**P4AB**	22.7		**P4AB**		27.6
**BI2a**	120.1	**P4PB**	29.0		**P4PB**		32.5
**BP2**	80.4	**M1OL**	44.2	42.7	**M1OL**	48.1	41.7
**BM1**	79.4	**M1IL**	41.0	34.9	**M1IL**	49.7	47.6
**BM3**	63.1	**M1AB**	40.1	40.8	**M1AB**	35.6	
		**M1PB**	41.1	32.3	**M1PB**	37.5	36.3
		**M2OL**	51.4	52.4	**M2OL**	61.7	65.0
		**M2IL**	51.1	49.2	**M2IL**	62.9	62.6
		**M2AB**	50.9	50.0	**M2AB**	46.5	46.0
		**M2PB**	50.6	50.8	**M2PB**	47.1	45.6
		**M3OL**	48.6	43.6	**M3OL**	74.0	73.4
		**M3IL**	47.2	44.4	**M3IL**	75.6	73.2
		**M3AB**	51.2	50.5	**M3AB**	47.8	45.4
		**M3PB**	43.7	43.6	**M3PB**	41.4	43.1

6 dental variables have been considered for upper and lower teeth: length (L) and breadth (B) for upper and lower incisors and canines, and outer length (OL), inner length (IL), anterior breadth (AB) and posterior breadth (PB) for the upper and lower premolars and molars (Table 1).

Literature data on fossil hippopotamuses from European Pleistocene sites and extant *Hipppopotamus amphibius* were considered [6, 9, 16, 63]. Fossil specimens of *Hippopotamus antiquus* housed in the University Museum of Earth Science, Department of Earth Sciences, Sapienza University of Rome (Ponte Molle, Vallinfreda, Monte Antenne) and PaleoFactory Laboratory, Sapienza University of Rome (Saticula and Bussi) were also included. Extant material of *Hipppopotamus amphibius* stored in the Comparative Anatomy Museum “Battista Grassi”, Sapienza University of Rome and PaleoFactory Laboratory, Sapienza University of Rome was also included. The biometric data published by Faure (1985) were not considered in this work because the measurements were taken differently from [16] and later studies.

Measurements were taken to the nearest 0.1 mm with a digital caliper.

A Principal Component Analysis (PCA) was conducted on the cranium of Tor di Quinto, Pleistocene and extant hippopotamuses (Table 1 in S1 File). Only a few cranial measurements of Pleistocene hippopotamuses are available in paleontological literature. In order to include the large number of specimens in the PCA, we selected eight cranial variables: HopA, HBA, BfM, BN, Botot, Boc, BTl and Bz.

Considering the limited number of specimens in the dataset, the values of the first component (PC1) have been chosen as independent variables in the ANOVA model (Analysis of Variance model) to explore possible differences among groups. The fossil from Cava Montanari was chosen as corner point, and therefore compared with other fossil and extant groups. We selected a significance threshold of 0.01. All statistical analyses were performed using the software R [64].

Standard boxplot was also carried out in order to explore the affinity of the Tor di Quinto specimen with other Pleistocene and extant hippopotamuses (see Table 2 in S1 File). We select only two variables: the LCN, the most important measurements to evaluate the cranial size of fossil hippopotamuses (not included in the PCA), and the M_3_OL, the most important tooth in fossil hippopotamuses to test possible size differences among fossil and extant taxa (the lower third molar is also the most easily recognizable in the lower molars).

Finally, the size of the upper and lower canines (antero-posterior diameter, [CL] vs. the lateral diameter [CB] of the upper and lower canines) of studied material was compared to that of extant specimens of *H*. *amphibius* to better define its sex, while for the age the method proposed by [65], based on the tooth wear, is applied (see S1 File).

### Sedimentological and geological results

#### Sedimentological implications from 3D model

Examining the texture of the historical photograph of Cava Montanari (Fig 3A), horizontal-bedded granular sediments (i,e, sands and pebbles) can be distinguished from the grass. As aforementioned, no information about the stratigraphical provenance was known. During the restoration work, sediments were found inside the cavities of the cranium and mandible, at the base of upper and lower teeth and partially encrusted on the outer side of the left hemimandible. These sediments are characterized by small pebbles (from 0.5 to 5 cm) and yellowish sands (Fig 1C–1F in S1 File). Observing the outer surfaces of the mandible bone, several depressions are observable (Fig 5). In order to better define these bone modifications, a 3D reconstruction of the left hemimandible of MPUR/149 has been realized, being this portion the area where these depressions were mostly concentrated. The 3D model allowed the investigation of the small depression clearly noted on the outer surface of the left hemimandible, more evident when the grazing light was set (Fig 5B, 5C). Thus, a depth-map was applied to test the dimension and the depth of these pits. The majority of the depressions was depth between 1.0 and 2.7 cm. As can be seen in Fig 5, their 2D profile, in most of the cases, was oval or circular (red/pink spots). The outline and depth of these depressions matches the pebbles found inside the cavities (Fig 1C–1F in S1 File). These results confirmed that probably the *Hippopotamus* skull was collected in a sandy deposit with pebbles ranging from 0.5 to 5 cm.

#### Petrographic results

The compositional signatures of sand samples recovered from the *Hippopotamus* skull show a feldspatho-litho-quartzose signature (Q_55_F_19_L_26_) whereas sand from the FCZ, VGU and VTN formations show a feldspatho-quartzo-lithic signature (Q_35_F_22_L_43_) (Fig 7).

Overall monomineralic grains are abundant (>55%) and include mainly quartz and feldspar (plagioclase and k-feldspar) grains, and minor micas (muscovite, biotite), and opaque and non-opaque (pyroxenes) heavy minerals. Lithic fragments are common (<45%) and include mostly sedimentary lithic fragments (carbonate, chert, siltite and argillite lithic fragments), with minor low-rank metasedimentary and volcanic lithic fragments.

The main grain types are those that, in different percentages, [54–56] have recognized in the Modern Tiber River sand which show a feldspatho-quartzo-lithic signature (Q_25_F_15_L_60_).

### Systematic paleontology

Class Mammalia Linnaeus, 1758

Order Artiodactyla Owen, 1848

Family Hippopotamidae Gray, 1821

Genus *Hippopotamus* Linnaeus, 1758

*Hippopotamus amphibius* Linnaeus, 1758

### Morphological description

#### Cranium (Fig 8A–8E)

MPUR/V 149 is a well-preserved skull, proportionally short and massive. The anteriormost portion of the nasals and right maxillae is damaged. The nasals are long and extend posteriorly up to the level of the middle part of the orbit. By tracing a longitudinal plane, the outside border of the right canine alveolus passes inside of the zygomatic arch at the level of the contact between frontal-temporal processes of the zygomatic bone. The zygomatic arches are broad and triangular in shape. The temporal ridges are short and few marked and converge caudally in a long and not prominent sagittal crest, which in turn, caudally merges to a well-developed, but not uplifted, nuchal crest. The temporal fenestrae are long.

**Fig 8:**
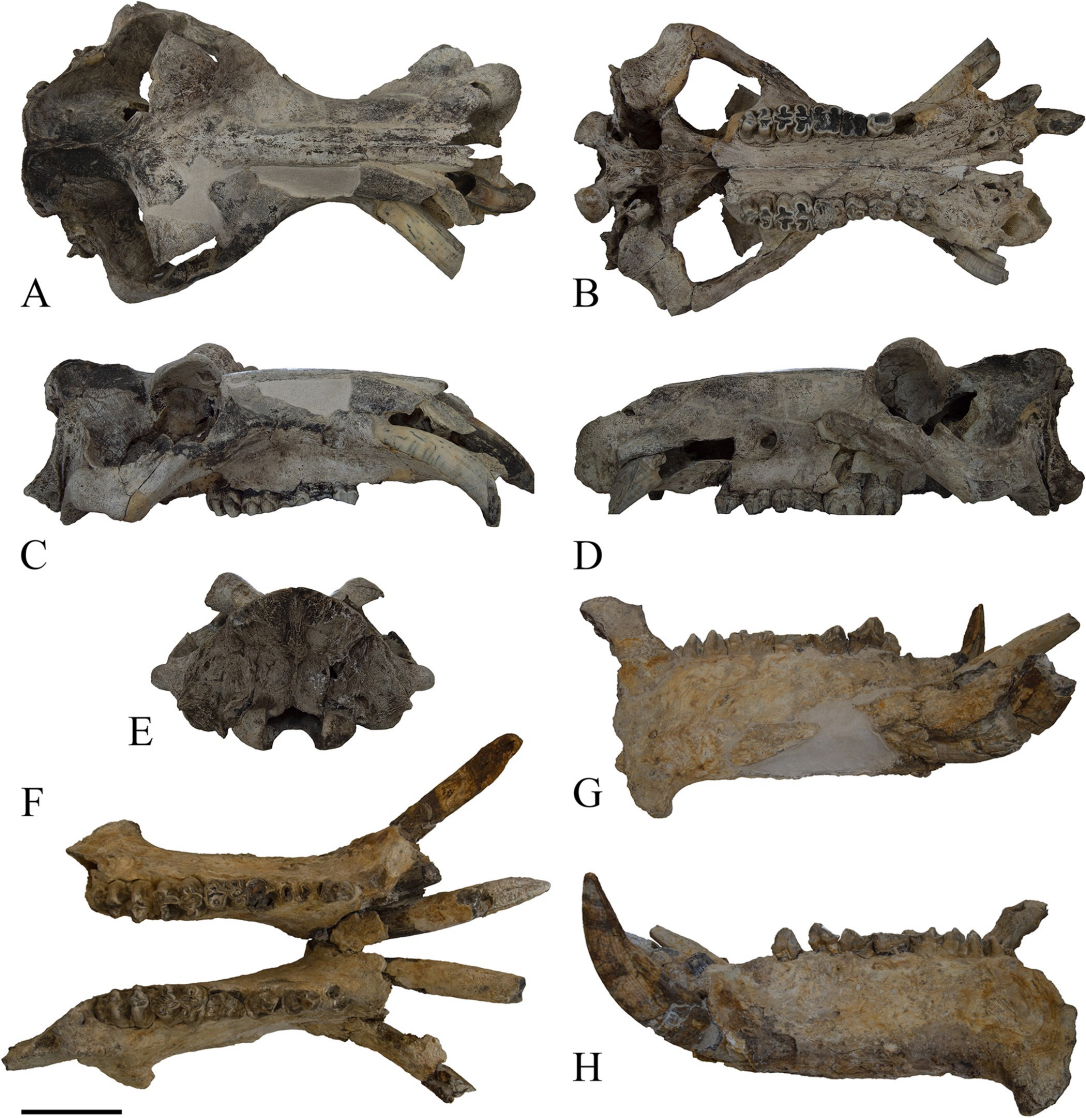
The skull of *Hippopotamus amphibius* of Cava Montanari. Cranium in dorsal (A), ventral (B), right lateral (C), left lateral (D) and posterior (E) views. Mandible in occlusal (F) right lateral (G) and left lateral (H) views. Scale bar 10 cm.

The nasal profile is almost parallel to the masticatory plane and converges anteriorly, even if it is slightly convex in the middle part, and it forms a wide-open angle with the parietal profile in lateral view. The infraorbital foramen is oval in shape, elongated antero-posteriorly, and it opens at the level of P^3^-P^4^. The zygomatic arch is robust and it emerges at the level of M^2^. The acustic meatus opens in the posterior portion of the temporal process of the zygomatic arch, it shows three main sulci dorso-ventrally oriented, a rugosity of the bone and a small opening of circular shape. The orbits are not elevated if compared to parietal and nasal profiles. The occipital profile is roughly vertical, and the occipital condyles do not protrude posteriorly. In posterior view, the occipital profile is semicircular in shape, with a marked crest-like rugosity in the middle part of the occipital bone, which connects the foramen magnum to the dorsal margin of the nuchal crest.

In ventral view, the first incisive line of the premaxillaries is projected anteriorly, displaying a well-developed notch between I^2^ and C^x^. The dental row is quite straight, and the maxillary profile is slightly arcuated at the level of the P^3^ and P^4^. The premaxillaries display two short palatine fissures, approximately oval in shape and elongated antero-posteriorly. The suture between premaxillaries and palatal bone emerges at the level of the posterior border of canine. The mandibular fossa is wide and it occupies nearly all the portion of the temporal bone in ventral view. Only the left tympanic bulla is partially preserved, which shows a large foramen in the postero-lateral corner.

#### Upper teet

The skull preserves the left C^x^_,_ P^2^, P^3^, P^4^, M^1^, M^2^, M^3^ and the right I^1^, I^2^, C^x^, P^3^, D^4^, M^1^, M^2^ and M^3^. The specimen displays an upper four deciduous molar in the right dental row. The D^4^ shows an extremely worn crown, hence limiting the description of morphological traits. The outline in occlusal view is semi-rectangular in shape, with the mesial portion of tooth smaller than the distal one. The presence of this tooth is interpreted as anomaly showed by the studied specimen, since in the left toothrow the normal configuration occurs.

I^1^ is the largest incisor teeth, with an oval section and an advanced wear of the crown. Its upper portion is broken.

I^2^ shows a circular section and advanced wear of the crown.

C^x^ displays a triangular section elongated labiolingually, and it shows a deep sulcus in the central portion of the teeth on the lingual side. The crown is an advanced stage of wear, complicating the observation of the enamel ridges and grooves on the tooth surface.

P^2^ possesses an asymmetric robust cusp projected mesially, with a slightly worn crown. The lingual cingulum is quite marked, whereas the labial, mesial and distal ones are absent.

P^3^ is similar to P^2^, but it is broader in the distal portion. The cusp displays a slightly worn crown. The distal and lingual cingula are quite marked, whereas mesial and buccal ones are absent.

P^4^ is the shortest premolar, and quite circular in shape in occlusal view. The main cusp, with a slightly worn crown, is not well-developed and it occupies the central portion of the tooth. Distal, lingual and mesio-lingual cingula are well marked, whereas the buccal one is missing.

M^1^, rectangular in shape in occlusal view, shows a crown in an advanced degree of wear. This prevents to detect the morphological features of the main cusps. Mesial and labial cingula are well marked.

M^2^, trapezoidal in shape in occlusal view, displays a moderately worn crown. The two mesial cusps, paracone and protocone, show a comma-shaped pattern, whereas the posterior ones, metacone and metaconule, are trefoil-shaped. The cingulum is quite marked along the mesial, distal and labial margins of the tooth, whereas it is almost absent along lingual margin. Pillars are absent and the transverse valleys, both on lingual and labial sides, are U-shaped.

M^3^, trapezoidal in shape in occlusal view, shows a slightly worn crown. The paracone is of a trefoil-shaped pattern, whereas the protocone, the metacone and metaconule are comma-shaped. The mesial and distal cingula are well marked, whereas the labial and lingual ones are quite absent. In the central portion of the distal cingulum, a stylid-like (distostyle) is observed. The lingual outlet of the transverse valley is U-shaped, whereas the labial profile is V-shaped.

#### Mandible (Fig 8F–8H)

The mandible MPUR/V 149 is in a good state of preservation. In general, the anteriormost portion of the mandible is quite fragmentary, but incisors and canines are preserved. The coronoid processes, the condylar processes and the distal portions of the mandibular ramus are not preserved.

In lateral view, the basal profile of the horizontal ramus is relatively straight, and the corpus is high.

The mesial border of the masseteric fossa is at the level of the entoconid and hypoconid cusps of the second molar and it matches with the mesial border of the angular process.

In occlusal view, the general aspect of the mandible is short and massive, and the outline of the tooth row is quite straight.

#### Lower teeth

The mandible preserves the left I_1_, I_2_, C_x_, P_2_, M_1_, M_2_ and M_3_, and the right I_1_, C_x_, P_3_, P_4_, M_1_, M_2_ and M_3_.

I_1_ is the largest incisor teeth, with an oval section and a moderate wear of the crown.

I_2_ shows a semicircular section and the crown of the only preserved (the left) is broken at about 2 centimeters above the incisive bone of the mandible. In the middle part of the mesial portion of the tooth a deep sulcus is present.

C_x_ is mesiodistally elongated with a triangular section elongated labiolingually. The crown is in a moderate stage of wear. A large, deep and parallel groove can be observed along the lingual and labial sides, whereas a shallow sulcus is present in the central portion of the tooth on the lingual side.

P_2_ possesses a symmetric robust cusp, with a slightly worn crown. A marked cingulum runs all along the tooth. A robust pilar is present on the lingual side, at approximately half of the tooth.

P_3_ possesses an asymmetric cusp projected mesially, which displays a slightly worn crown. All the cingula are well marked. A robust pilar can observed in lingual side at the level of the main cusp.

P_4_ is similar to P_3_, but slightly larger. The main cusp shows a slight wear of the crown. A marked cingulum runs all along the tooth, especially marked in the distal portion.

M_1_, rectangular in shape in occlusal view, shows an advanced worn crown. The protoconid and the metaconid are trefoil-shaped. The advanced wear of the teeth prevents to detect the morphological features of the main distal cusps (hypoconid and entoconid), the outlets of the lingual and labial transverse valleys and the presence/absence of the cingula. A small pilar is present in the middle part of the left M_1_, near the lingual side.

M_2_ is rectangular in shape in occlusal view, and it shows a slightly worn crown. The entoconid is comma-shaped, whereas the protoconid, the entoconid and the hypoconid are trefoil-shaped. The lingual and labial transverse valleys are V-shaped. Mesial and distal cingula appear as cusped-like, whereas the labial and the lingual ones are present and quite marked only at the level of transverse valleys.

M_3_ is the longest molar tooth. It displays a weakly worn crown. The metaconid, the protoconid and the hypoconid are trefoil-shaped, whereas the entoconid shows a comma-shaped pattern. The postmetacristid is in contact with the prehypocristid. The hypoconulid would seem cruciform-shaped, but the crown is not worn. The labial and lingual outlets of the transverse valleys are V-shaped. Only the mesial cingulum is present and marked.

Two small pillars are present along the labial margin: the first between the protoconid and the hypoconid and the second between the hypoconid and the hypoconulid.

### Morphological comparison

The cranium of Cava Montanari is proportionally short and of massive morphology, the outside border of the canine alveolus does not protrude more than the zygomatic process, the sagittal crest is long and not relatively prominent, the temporal fenestrae are elongated, the occipital profile is vertical with not prominent occipital condyles, the nuchal crest is not uplifted, the orbits are not elevated and the nasal profile is almost parallel to masticatory plane and convergent anteriorly.

**Fig 9:**
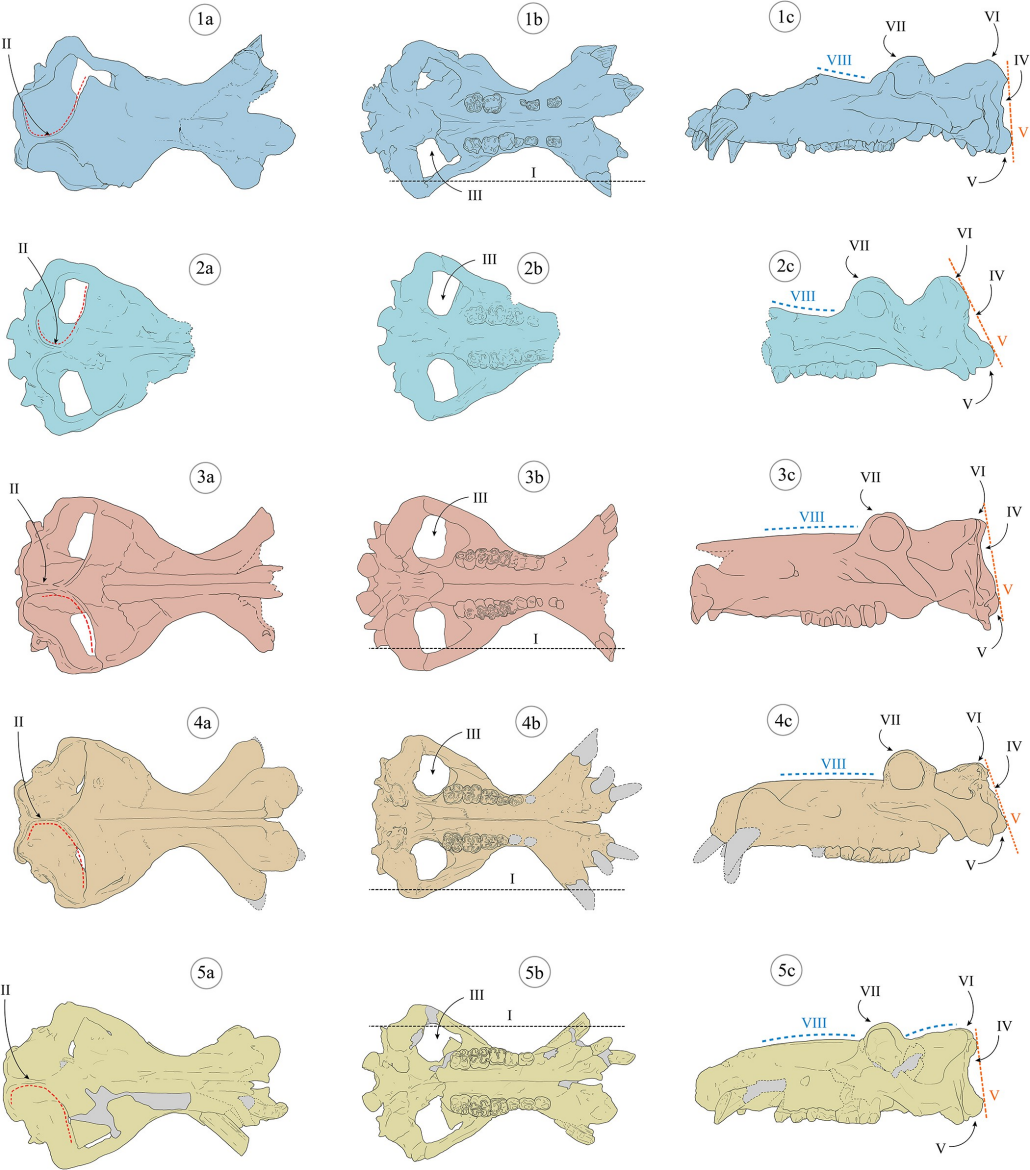
Cranial morphological features of selected extant and fossil hippopotamus specimens. The following numbers indicate the main morphological features proposed in literature used to discriminate *Hippopotamus antiquus* and *Hippopotamus amphibius* (as reported in Table 2). Colours: blue—*H*. *antiquus* from Figline (Upper Valdarno, 1a-1c, modified from [65]); light blue—*H*. *antiquus* from La Maglianella (2a-2c, modified from [15]); red–*H*. *amphibius* from Barrington (3a-3c, modified from [65]); orange—extant specimen of *H*. *amphibius* (4a-4c); yellow—*H*. *amphibius* from Cava Montanari (5a-5c). Cranium in dorsal (a), ventral (b) and left lateral (c) views.

All these characters align the *Hippopotamus* of Cava Montanari with extant and fossil *H*. *amphibius* ([8, 16]; Fig 9; Table 2). Different cranial characters are observed in the extinct *H*. *antiquus* ([8, 16], Table 2, Fig 9).

**Table 2:** Main diagnostic cranial and mandibular characters proposed in literature for fossil and extant hippopotamuses.

Number as illustrated in the Fig 9	Cranial diagnostic characthers	Species		Reference
		*Hippopotamus antiquus*	*Hippopotamus amphibius*	
**I**	Outside border of the canine alveolus	Protuding than the zigomatic process	Not protuding than the zigomatic process	Caloi et al. (1980)
**II**	Sagittal crest	Short and prominent	Long and not prominent	Caloi et al. (1980); Mazza (1995)
**III**	Temporal fenestrae	Short	Long	Mazza (1995)
**IV**	Occipital	Vertical	Vertical	Caloi et al. (1980); Mazza (1995)
**V**	Occipital Condyles	Prominent	Not prominent	Caloi et al. (1980); Mazza (1995)
**VI**	Nuchal crest	Uplifted	Not uplifted	Mazza (1995)
**VII**	Orbit	Elevated	Not elevated	Caloi et al. (1980); Mazza (1995)
**VIII**	Nasal profile	Slopes anteriorly upwards and forms a small angle with parietal profile	Parallel to masticatory plane and converges anteriorly	Caloi et al. (1980); Mazza (1995)
**Number as illustrated in the Fig 10**	**Mandible diagnostic characthers**			
**I**	Horizontal ramus	Long and slender wtih flat or concave basal profile	High with a convex basal profile	Caloi et al. (1980); Mazza (1995)

**Fig 10:**
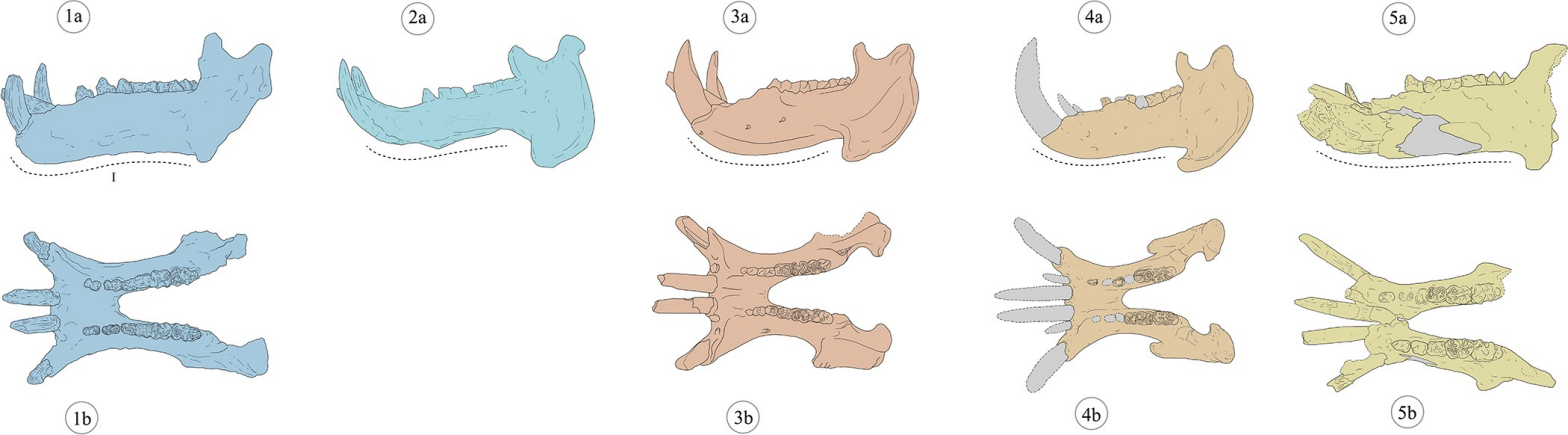
Mandibular morphological features of selected extant and fossil hippopotamus specimens. The following numbers indicate the main morphological features proposed in literature used to discriminate *Hippopotamus antiquus* and *Hippopotamus amphibius* (as reported in Table 2). Colours: blue—*H*. *antiquus* from Figline (Upper Valdarno, 1a, 1b, modified from [65]); light blue—*H*. *antiquus* from La Maglianella (2a, modified from [15]); red—*H*. *amphibius* from Barrington (3a, 3b, modified from [65]); orange–extant specimen of *H*. *amphibius* (4a, 4b); yellow—*H*. *amphibius* from Cava Montanari (5a, 5b). Mandible in left lateral (a) and occlusal (b) views.

For the mandible, the only feature useful for a specific distinction between *H*. *antiquus* and *H*. *amphibius* is the morphology of the horizontal ramus in lateral view. In the specimen from Cava Montanari the corpus is high but relatively straight, an intermediate state than what described for the two species.

No dental morphological characters have been proposed for discrimination between the considered species, except for the development of ridges and grooves on the surface of upper and lower canines. Following [8, 66], *H*. *antiquus* possesses fairly parallel ridges and grooves along the lateral surfaces, while these are convergent forward in *H*. *amphibius*. By contrast, [16] noted that the arrangement of the enamel ridges and grooves is quite variable, concluding its scarce value for species identification purposes. In lower canines of the studied skull, the ridges and grooves are prominent and convergent toward the end of the tooth along the lingual and labial sides. This character cannot be observed in the upper canine due to the advanced tooth wear.

### Biometric comparison

**Fig 11:**
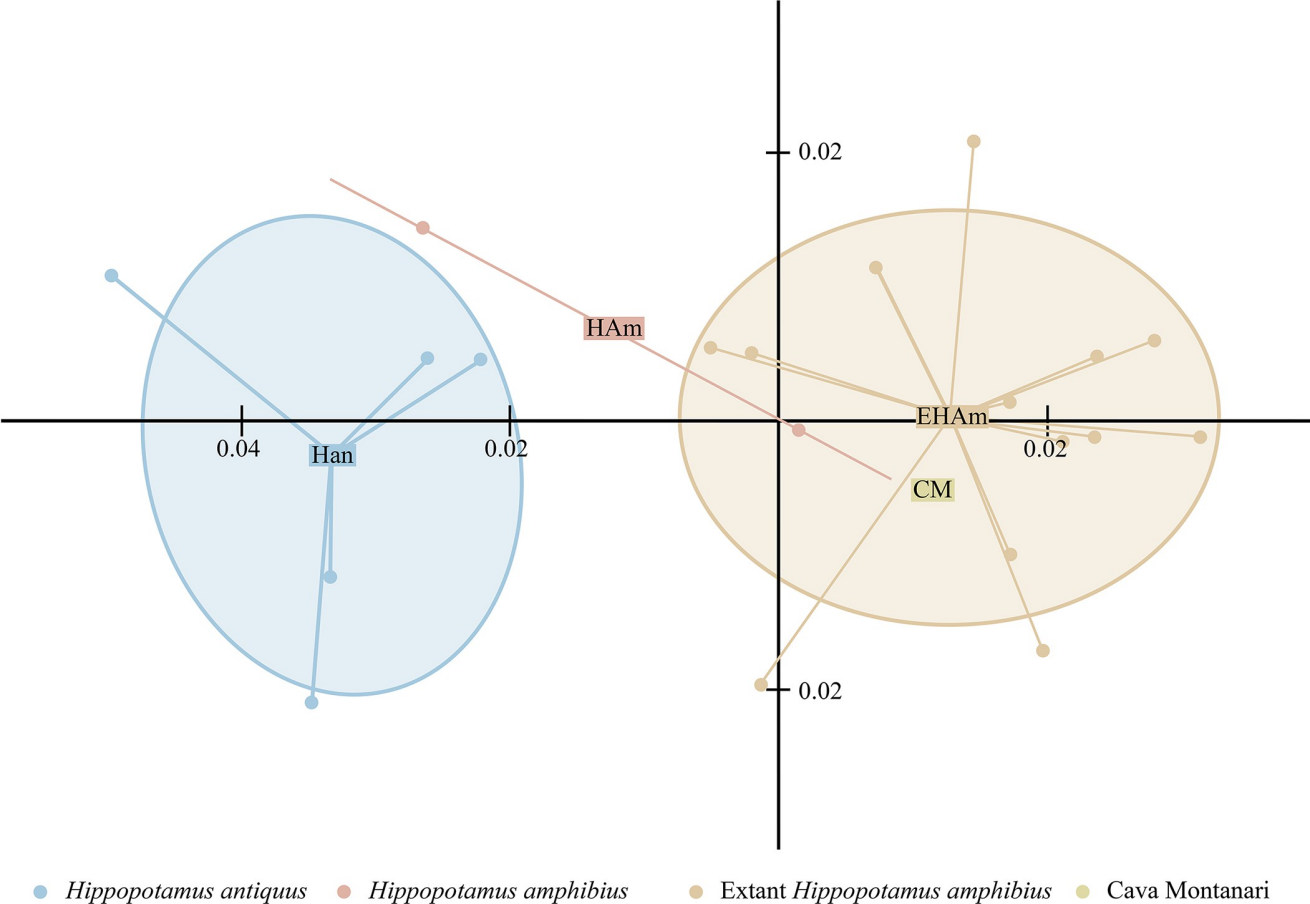
Scatter plot of the first two principal components of Principal Component Analysis (PCA).

The two first Principal Components (PCs) from the Principal Component Analysis performed with the cranial biometric variables maintain the 80% of the total variance (Fig 11; Fig 3 in S1 File). The scatter diagram shows the biometric differences in the skulls of the two main groups considered: i) *Hippopotamus antiquus* and ii) extant *Hippopotamus amphibius* and Cava Montanari. The sample of Barrington, the only representative of the fossil *Hippopotamus amphibius*, partially falls in the variability of the second group (Fig 11).

The first component (PC1) accounts for 66.2% of the total variance and all the variables positively contribute to it, with the breadth of the nuchal crest (BN), the breadth across the occipital condyles (BOc) and the zygomatic breadth (BZ), opisthion-akrocranion height (HOpA), basion-akrocranion height (HBA) and otion-otion breadth (Botot), being slightly more important than others variables (Fig 4 in S1 File). The second component (PC2) explains the 13.8% of the total variance, with most of the contribution given by the breadth between the temporal lines (BTl) and the breadth of the foramen magnum (BFm) (Fig 5 in S1 File).

PC1 separated the groups based on size, with larger specimens on the left and smaller specimens on the right. The PC1 was subject to normality distribution verification using Shapiro Test. The results of the ANOVA model applied on PC1 reveals that the Cava Montanari specimen differs from *H*. *antiquus* (p.value < 0.01), while is similar to fossil and extant *H*. *amphibius* (p.value > 0.01).

The value of the canine alveolus-nuchal crest length (LCN) of Cava Montanari cranium fell within the range of variation of the extant *H*. *amphibius*, and it is shorter than *H*. *antiquus* and fossil *H*. *amphibius* (Fig 6 in S1 File).

In the boxplot of the lower third molar (Fig 7 in S1 File), the value of Cava Montanari is slightly longer than those of the fossil *H*. *amphibius*, and it reaches the lower range of variation of *H*. *antiquus* and extant *H*. *amphibius*. As aforementioned, the lower third molar of the studied specimen is just erupted, with a quite unworn crown. This could explain the apparent long value for the tooth.

## Discussion

### Sedimentological context of *Hippopotamus* skull

The sediments found inside the cavities of the MPUR/V 149 offer the possibility to characterize the geological features of the deposit where this skull was collected. One of the aspects that could represent a limit in our approach is to have found sediments mainly inside cavities of the cranium and the mandible. From a taphonomic point of view, the sediments inside these cavities might have been selected by the size of the opening of the missing part of the bone. Looking at the right hemimandible during the restoration work (Fig 1C in S1 File), it is quite evident that the size of the pebbles is much less when compared to the opening resulting by a broken of the bone. Furthermore, after removing the colour that masked the bone surface during the restoration work, depressions with oval or circular outline were observed especially on the outer side of the left hemimandible. The digital investigation of the outer surface of the left hemimandible shows clearly the presence of these depressions, whose size ranging from 0.5 to 5 cm, generally with a 1 to 2.6 cm depth (Fig 5). This indicates that during the depositional process small pebbles encrusted the bone of the MPUR/V 149, affecting the preservation of the outer surfaces. All these considerations support that MPUR/V 149 fossil was collected in a sandy deposit with small pebbles (ranging from 0.5 to 5 cm).

The petrographic data obtained to characterize sediment composition recovered inside the cavities of the *Hippopotamus* skull were compared with sand detrital modes from the modern and ancient deposits sourced by the Tiber River system to obtain direct information about the sediment provenance and the stratigraphic context of the enclosing sedimentary unit.

Results of the analyses suggest that the sediment recovered from the *Hippopotamus* skull was transported by an ancient equivalent of the Tiber River whose modern sand is composed of a mixture of quartz, feldspar, mica, dense minerals and lithic fragments, the latter including mostly sedimentary and igneous rock fragments [53, 54]. The sedimentary lithics, which are typically composed of carbonate, chert grains and siliciclastic rock fragments, reflect the sedimentary input from the carbonate successions of the upper and middle Tiber drainage basin, while the igneous lithic fragments are typically of volcanic in origin and suggest the influence of volcanic activity in the middle and downstream regions [53, 54, 67]. The sediment signature of the modern Tiber River sand is summarized in the compositional biplot of Fig 7, which defines two main compositional fields characterizing an overall sedimentaclastic signature in the upper and middle drainage basin sand and a volcanoclastic signature for the downstream sand. The sediment signature of the ancient deposits sampled from the FCZ, VGU, and VTN formations and from the *Hippopotamus* skull matches the sedimentaclastic modern signature suggesting that the paleo-Tiber River was primarily eroding the sedimentary successions of the central Apennines. This indicates that volcaniclastic rock units were not a significant source of sediment during that time and that the sediment being carried by the paleo-Tiber River did not contain significant amounts of volcanic lithic grains.

**Fig 12:**
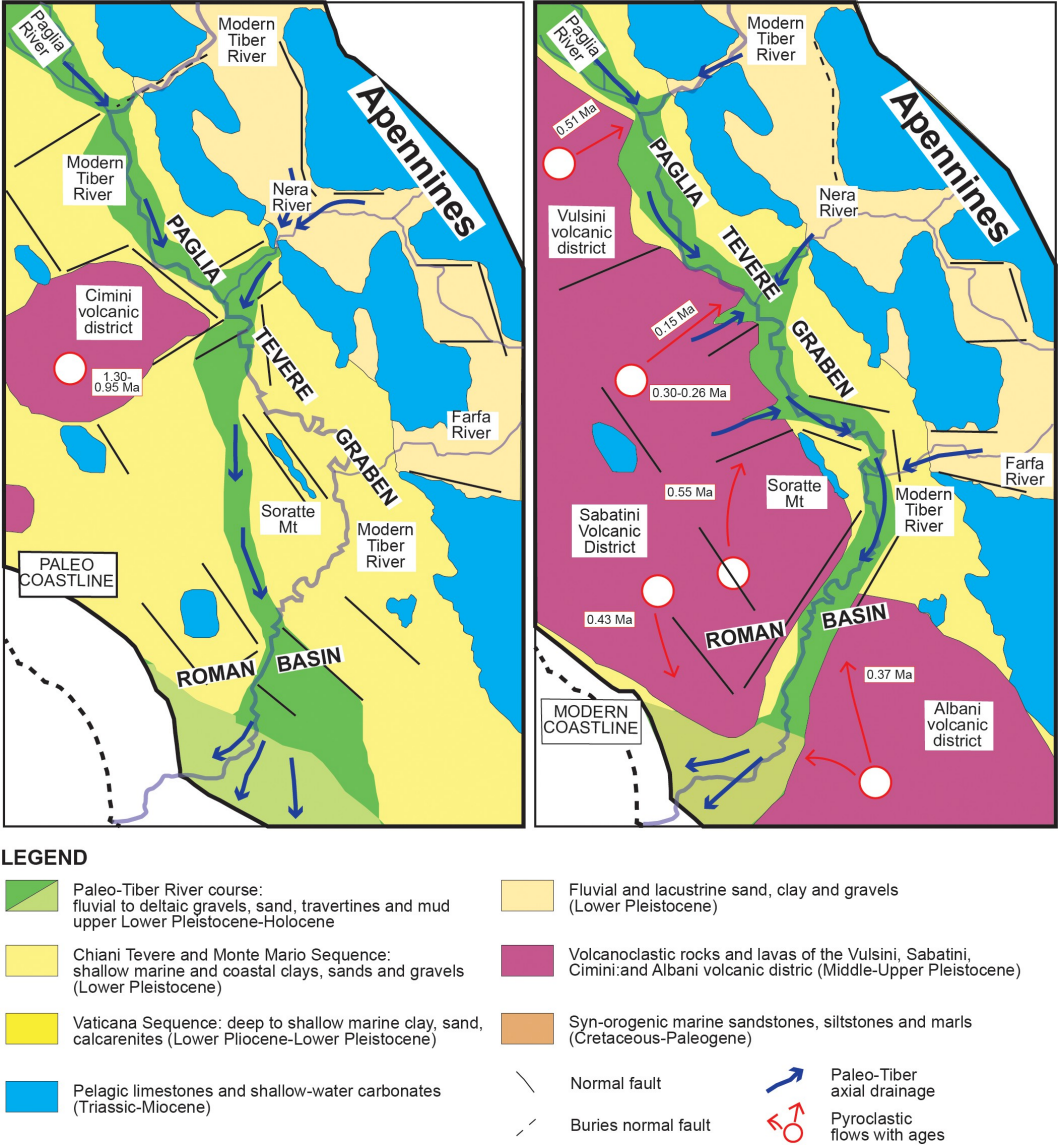
Paleogeography of the middle Tiber Valley Basin during the uplift dominated phase (latest Early Pleistocene-Late Pleistocene, from 1.3 to 0.1 Ma). To the left, location of the paleo Tiber fluvial system within the Paglia Tevere graben developed at the west Apennines foothill (in green with axial drainage). To the right, volcanic activity of the Vulsini Mts., Vico, Sabatini Mts. and Albani Hills Volcanic Districts (in pink) and their impact on the Tiber fluvial drainage system. The main pyroclastic flows are dated and reported with red arrows (redrawn and modified after [66]).

Indeed, before the onset of the volcanic activity, the differential uplift rates between the raising Apennines chain and the subsiding Paglia-Tevere Graben during the Early Pleistocene, resulted in enhanced detrital influx of coarse-grained carbonaticlastic fluvial sediments from the ancient tributaries draining the carbonate succession of the central Apennines [67] (Fig 12). As the regional uplift continued throughout the rest of the Quaternary, the transverse ancient rivers draining the Mesozoic pelagic carbonates succession of the central Apennines (e.g., paleo-Nera and paleo-Farfa rivers) connected directly to the newly formed Tiber River system and provided the Paglia-Tevere Graben with coarse-grained fluvial sediment [67–69]. Subsequent erosional and depositional phases associated with the main glacio-eustatic fluctuations resulted in the deposition of fluvial terraced deposits and the partial cannibalization and redeposition of the older tributaries and Tiber River’s deposits. Following the paroxims of the Vulsini Mts., Vico, Sabatini Mts. and Albani Hills Volcanic Districts in the Middle–Upper Pleistocene [70–75] the downstream fluvial system reorganized and the recycling of volcaniclastic units became predominant.

### Chronostratigraphic framework

Further observations were carried out to help identify the stratigraphic units hosting the skull specimen and to obtain indirect indication about the maximum age of the sedimentary deposits by comparing the sand composition between the *Hippopotamus* skull, and the FCZ, VGU, and VTN sand deposits. The LmLvLs ternary plot and the compositional biplot of Fig 7 show similar lithic fragment composition between the skull sediment and the VGU sand, partial overlap between the skull sediment and the FCZ sand, and different composition between the skull sediment and the VTN sand deposits. These results combined with stratigraphic observation and the spatial distribution of the sedimentary units in the proximity of the excavation site, suggest that the VGU formation can be considered the most likely unit originally hosting the *Hippopotamus* skull, with an age spanning between 560 and 460 ka.

The overall lower concentration of lithic fragments in the sand samples from the hippo, FCZ, VGU and VTN deposits with respect to modern sand might depend on a variety of factors including dilution processes and sediment grain-size variability. Lithic-rich modern sand was sampled from tributary rivers and at the intersection between the tributaries and the main Tiber River trunk in the proximity of the source rocks and thus experienced relatively limited transport and dilution. Instead, ancient sand from FCZ, VGU and VTN deposits were sourced by the downstream equivalent of the Tiber River system and experienced a longer-distance transport and a dilution of the lithic-fragment signature. Also, the sediment recovered from the *Hippopotamus* skull shows a lower proportion of lithic fragments that can be explained with the effect of hydraulic sorting during fluvial transport or postdepositional *in situ* weathering processes. In particular, the relatively finer-grained sand recovered from the skull show higher monomineralic grains proportion with respect to the coarser grained lithic-rich sand. These grain-size dependence in composition can be ascribed to different transport modes (e.g., suspended-load versus bedload sand). In this scenario, samples collected from fluvial bedload sand from the FCZ, VGU and VTN deposits will be virtually enriched in coarse-grained lithic fragments types.

*In situ* weathering potentially removes provenance information, reducing correlation potential of petrographic signatures [54]. Carbonate detritus is susceptible to chemical weathering which could reduce its percentage as lithic components through dissolution and alteration in outcrop. The role of postdepositional weathering in reducing the carbonate lithic fraction within the Tiber ancient sand has been already discussed in [54].

### Taxonomic attribution and implications for *H*. *amphibius* dispersal

The taxonomic attribution of the skull from the Tor di Quinto area to *Hippopotamus amphibius* has never been questioned (e.g., [8, 16, 28]) and is clearly supported by our results. The *Hippopotamus* of Cava Montanari displays cranial features that perfectly adheres to the morphology considered diagnostic for *H*. *amphibius* [8, 16]. The results of the PCA also underlines the distinction between *H*. *antiquus* and *H*. *amphibius*, and the placement of MPUR/V 149 within the morphospace occupied by *H*. *amphibius*. However, it is worth mentioning that only a few cranial traits permit a clear distinction between *H*. *antiquus* and *H*. *amphibius* (Table 2), whereas others are shared between the two species or prone to sex or age-related variation.

Following [8], for example, *H*. *antiquus* shows higher orbits than those of *H*. *amphibius*, but old individuals of extant hippo rarely display elevated orbits. [8] also suggested that diagnostic cranial morphologies are only valid for species identification if they are all concurrently observed together on the same specimens. Three of these features observed in *H*. *amphibius* are: a vertical occipital bone, not posteriorly prominent occipital condyles and a parallel nasal profile to masticatory plane with an anterior convergence. These characters are all detected in the cranium of Cava Montanari. In the mandible, the morphological similarity between the two species is even more evident, being the profile of the horizontal ramus the only reliable feature for taxonomic discrimination. Excluding the canines, dental remains show conservative morphologies, indistinguishable between species.

For the canines, paleontologists held contrary opinions on the validity of the configuration of the enamel ridges and grooves on the surface as diagnostic character (considered valid by [8, 65], but not by [16]). In the skull of Cava Montanari, the lower canines show prominent and convergent enamel ridges, trait frequently observed in fossil and extant *H*. *amphibius* [8, 16]. [16] argued that the two distinct morphotypes (prominent and convergent enamel ridges and parallel development of enamel ridges) are observed with the same frequency in *H*. *antiquus*. The author, however, reported no detailed information on the distribution of these morphotypes in the fossil record. It is worth stressing that [16] only accepted a presence of *H*. *amphibius* since the early Late Pleistocene, basically attributing all Middle Pleistocene specimens to *H*. *antiquus* (= *H*. *tiberinus*). For other authors, the earliest dispersal of the modern hippopotamuses occurred ca. 500 ka (MIS 13, e.g., [8, 6, 17, 22, 29]. These contrasting taxonomical views pose difficulties in recognizing on what specimens the considerations on canine morphology have been based, and in turn to evaluate the reliability of the characters proposed by [66]. For clarifying this aspect, the European Middle Pleistocene fossils of hippopotamuses should be revised.

Our results highlight that the skull of European Pleistocene hippopotamuses preserves reliable diagnostic characters for the distinction between *H*. *antiquus* and *H*. *amphibius*, but relevant fossils are quite limited. More common are isolated teeth and postcranial bones, which, however, do not allow exhaustive taxonomic discrimination. As aforementioned, the dental remains do not show significant morphological differences, and commonly their identification is based on size (with *H*. *antiquus* larger than *H*. *amphibius*). For postcranial bones, even if not included in this work, a preliminary analysis for the discrimination between these two species was published by [16], but only a few works attempted to explore and to expand the knowledges for unambiguous cut-criteria [9, 22, 23, 61, 63]. Their results are not conclusive, leaving skepticism on the use of these features for specific identification.

The Cava Montanari fossil is one of the few skulls of the Middle Pleistocene in Europe. The revision of the historical geological documentation indicates that the skull was deposed between 560 and 460 ka, representing the earliest unequivocal occurrence of *H*. *amphibius* in Europe. Reassessing the age of the Cava Montanari fossil pushes to critically reconsidered the *H*. *antiquus*-*H*. *amphibius* transition in Europe. More specifically, the Middle Pleistocene specimens dated around 500–400 ka are mainly represented by isolated teeth and postcranial elements that, as already widely discussed, are of poor taxonomic value [21]. In this context, investigating the disappearance of *H*. *antiquus* and the possible coexistence between it and early representatives of *H*. *amphibius* would be a relevant topic for future research.

Based on the Cava Montanari record, the earliest dispersal of *H*. *amphibius* occurred around 500 ka, during the last part of the Early-Middle Pleistocene Transition (EMPT). The EMPT (ca. 1.2–0.4 Ma) represents a period of substantial change in in Earth’s climate system, with a progressive and yet not gradual increase in the amplitude of climate oscillations occurred between the late Early and the early Middle Pleistocene [76–78]. The climate changes were accompanied by several bioevents denoting a renewal of large mammal faunas, resulting in the identification of subsequent European Land Mammal Ages (ELMA; see [79], for discussion), the Epivillafranchian (ca. 1.2–0.8 Ma), which witnessed the coexistence of late Villafranchian holdovers with several newcomers and the Galerian (ca. 0.8–0.4 Ma), featuring the spread in Europe of several still living species, e.g., *Cervus elaphus*, *Sus scrofa* and *C*. *crocuta*, and of the Acheulean technocultural complex [24, 80–90]. Nonetheless, the pace of the turnover, the synchronicity between bioevents, the related biochronological correlations and nomenclature, and the precise relationships between climatic, environmental, and faunal changes are hotly debated (e.g., [91–93]). In this regard, providing a robust age for the earliest dispersal of the extant species *H*. *amphibius* adds an important piece of information. The occurrence of Cava Montanari confirms that modern hippopotamuses were already present in Europe around 500 ka, that is, shortly before the Mid-Brunhes Event (ca. 424 ka; MIS 12–11 transition), an event that is taken to correspond to the end of the EMPT. From a climatic perspective, the Mid-Brunhes Event marks the consolidation of the glacial cycles ruled by a 100 kyr periodicity, the longest cyclicity recognized during the Quaternary. Traditionally, the beginning of the Aurelian, the Mammal Age following the Galerian, was placed at ca. 325 ka (MIS 9, [79]), based on the first appearance of *Canis lupus*, *Megaloceros giganteus* and *Ursus spelaeus*. In the last decades, however, the earliest dispersal of these taxa has been recognized to be older (MIS 13 or MIS 11) [27, 94, 95]. Furthermore, during the end of the EMPT, multiple key bioevents occurred in Europe, among which the spread of *Bos primigenius*, *Equus hydruntinus* and *Dama clactoniana* [27, 33, 96–98]. The appearance of *H*. *amphibius* in the European fossil record ca. 500 ka is another bioevent that can be added to this list, anticipating previous estimates.

### Paleoecology and the extinction of *H*. *amphibius* in Europe

Modern hippopotamuses have long been considered strict indicator of warm climatic conditions, but current data rather highlights their reliance on the presence of permanent body waters (e.g., [3, 6, 99, 100]). The climatic instability of the Middle Pleistocene likely influenced the distribution of hippopotamuses, since glacial stages were linked to drier climatic conditions and colder temperatures that, although probably not a limiting factor in itself, were responsible to freeze a larger part of water bodies; no freezing occurred during interglacial stages. It is not a coincidence that the southern regions of Europe (Iberian, Italian and Balkan Peninsulas) are considered glacial refugia for hippopotamuses species (e.g., [101]). These areas, even during a glacial stage, retained milder climates and a greater availability of permanent water bodies. Taking into consideration their ecological preference, after their dispersal into Europe during MIS 13, hippopotamuses might have survived in the South during MIS 12, an extreme and severe glacial stage [102]. It is likely that, during the late Middle Pleistocene, the geographical range of European hippopotamuses was affected by strong oscillations related to climatic changes, expanding into northern regions during interglacial stages and retreating to southern regions, with a patchier distribution, during the glacials.

In the Italian Peninsula, *H*. *amphibius* was thought to survive until MIS 4–3, based on the record of Grotta Romanelli (Apulia, southern Italy) (e.g., [20, 29]. Indeed, the fossils from level G (ISU3 *sensu* [103] were previously dated between 69.000 years BP and 40,000 ± 3250 years BP [104–106], but the chronostratigraphic reassessment of the infilling deposit of Grotta Romanelli revealed as level G can be referred to the early Late Pleistocene (MIS 5 [103]). Other putative younger Italian records of hippopotamuses come from Ingarano, Canale Mussolini (= Canale della Acque Alte), Grotta Guattari, Avetrana and Grotta dei Moscerini [107]. The fossils from Ingarano were found *ex situ*, near the main deposit [108]. No exact stratigraphic provenance is known for the specimens recovered at Canale Mussolini, and available dating support an age older than 54 ka for the lower levels of the succession [109, 110]. Hippopotamus remains were reported by [111, 112] from Grotta Guattari, whose sequence is bracketed between MIS 5 and MIS 3 [113–116], although with no precise information on their stratigraphic provenance or any description. Recently, also the site of Grotta dei Moscerini was constrained to the early Late Pleistocene (MIS 5, [115]). The faunal assemblage recovered from the karst infilling deposit of Avetrana was initially attributed to the early Late Pleistocene [117–119]. Always on the basis of the analysis of the mammal sample, the uppermost level of the succession (bed 8) was later referred to MIS 3 [120, 121]. An older age for the faunal remains recovered from the rest of the sequence (beds 1–7) was proposed by [122], who attributed these levels to the late Middle Pleistocene. In 2017, volcanic products were found at the base of the bed 8 and sampled for geochemical analysis [123]. The results of this work revealed an age of 132 ± 12 ka, confirming therefore a late Middle Pleistocene age for the beds 1–7. The hippopotamus remains were collected below the volcanic level ([123] and references therein).

In sum, recent research points to a probable disappearance of *H*. *amphibius* from the Italian Peninsula during the early Late Pleistocene (MIS 5).

## Conclusion

The hippopotamus skull MPUR/V 149 recovered from Cava Montanari, Tor di Quinto, a district of Rome (central Italy) and housed at Earth Science University Museum (MUST) of Sapienza University of Rome is here revised. Our morphological, biometric and statistical analyses reaffirm its attribution to *H*. *amphibius*.

The revision of the old paleontological, archeological and geological literature published during the 19^th^ and 20^th^ century on the Rome basin allowed us to shed light on the long-debated geographical provenance of MPUR/V 149, which is here assigned to be from Cava Montanari, a quarry opened along the Flaminia road, in the Tor di Quinto district.

This skull was restored in 2021, within a large restoration project on vertebrate exposed at the MUST. During these activities, original sediments were found inside the cavities of cranium and mandible of the studied specimen, which were sampled for sedimentological and petrographic analyses. The results, combined with the lithostratigraphic and synthemic units of the national geological cartography, revealed as the skull was collected from a deposit attributed to the Valle Giulia Formation, which age is between 560 and 460 ka.

Considering our results, MPUR/V 149 represents the oldest occurrence of *H*. *amphibius* in the European fossil record, reinforcing the hypothesis of a first dispersal of this taxon during the Middle Pleistocene.

## Supporting information

S1 File Text and figures. (DOCX)

S1 Table Cranial material considered in Principal Component Analysis (PCA). (XLSX)

S2 Table Measurements of the lower third molar considered in boxplot S7 Fig in S1 File. (XLSX)

S1 Appendix 3d model of the cranium of *Hippopotamus amphibius* from Cava Montanari. (PLY)

S2 Appendix 3d model of the right hemimandible of *Hippopotamus amphibius* from Cava Montanari. (PLY)

S3 Appendix 3d model of the left hemimandible of *Hippopotamus amphibius* from Cava Montanari. (PLY)
