# Supplementary material for: Reinforcing the idea of an early dispersal of Hippopotamus amphibius in Europe: Restoration and multidisciplinary study of the skull from the Middle Pleistocene of Cava Montanari (Rome, central Italy)
Source: PLoS One. 2023 Nov 22;18(11):e0293405. doi: 10.1371/journal.pone.0293405 (PMC10664965; doi:10.1371/journal.pone.0293405)
Supplement: S1 File — (DOCX) [file pone.0293405.s001.docx]

Supporting Information

Reinforcing the idea of an early dispersal of *Hippopotamus amphibius* in Europe: restoration and multidisciplinary study of the skull from the Middle Pleistocene of Cava Montanari (Rome, central Italy)

**Mecozzi Beniamino, Iannucci Alessio, Mancini Marco, Tentori Daniel, Cavasinni Chiara, Conti Jacopo, Messina Mattia Y., Sarra Alex, Sardella Raffaele**

Correspondence to**:** [Beniamino.mecozzi@uniroma1.it](mailto:Beniamino.mecozzi@uniroma1.it)

**S1 File. Text and Figures**

**This file includes:**

**Text**

**Figures S1-S7**

**Restoration work**

During the January 2021, the restoration work on the mammal fossils exposed in the Vertebrate Hall of the former Paleontological Museum of the Department of Earth Sciences, Sapienza University of Rome, started. The restoration activity falls within a large project that involved the museums of the Department of Earth Sciences, where the three museums of Geology, Minerology, and Paleontology were fused together, forming the University Museum of Earth Science (MUST).

The hippopotamus skull MPUR/V 149, object of this work, was figured for the first time by Fabiani & Maxia (1953), where the fossil had the same aspect compared with its state at the beginning of restoration activity.

The first step was to remove the color applied on the cranium and mandible, that give to the fossil a homogenous aspect. Removing this paint, it was possible to observe the original bone and the integrations realized in the past.

In the cranium, these integrations filled small bone portions damaged, as for example at the level of right nasals. Most important were the interventions on the right orbit and the anteriormost portion of the muzzle. In the first case, a mix of wax and gypsum was applied to reposition the right orbit bone in its original position. Nevertheless, a large quantity of material was used at the level of the frontal, making a quite flat surface among the orbits. The right anterior portion of the muzzle was also strongly reconstructed, following the original configuration of the left part.

In the mandible, a large part of the right labial corpus was damaged and covered by a mix of wax and gypsum. The anteriormost portion of the mandible was strongly restored, with the use of the metal support for keeping connected the left and the right branches. This masked the original morphology of the mandible. The right lower incisor was also reconstructed.


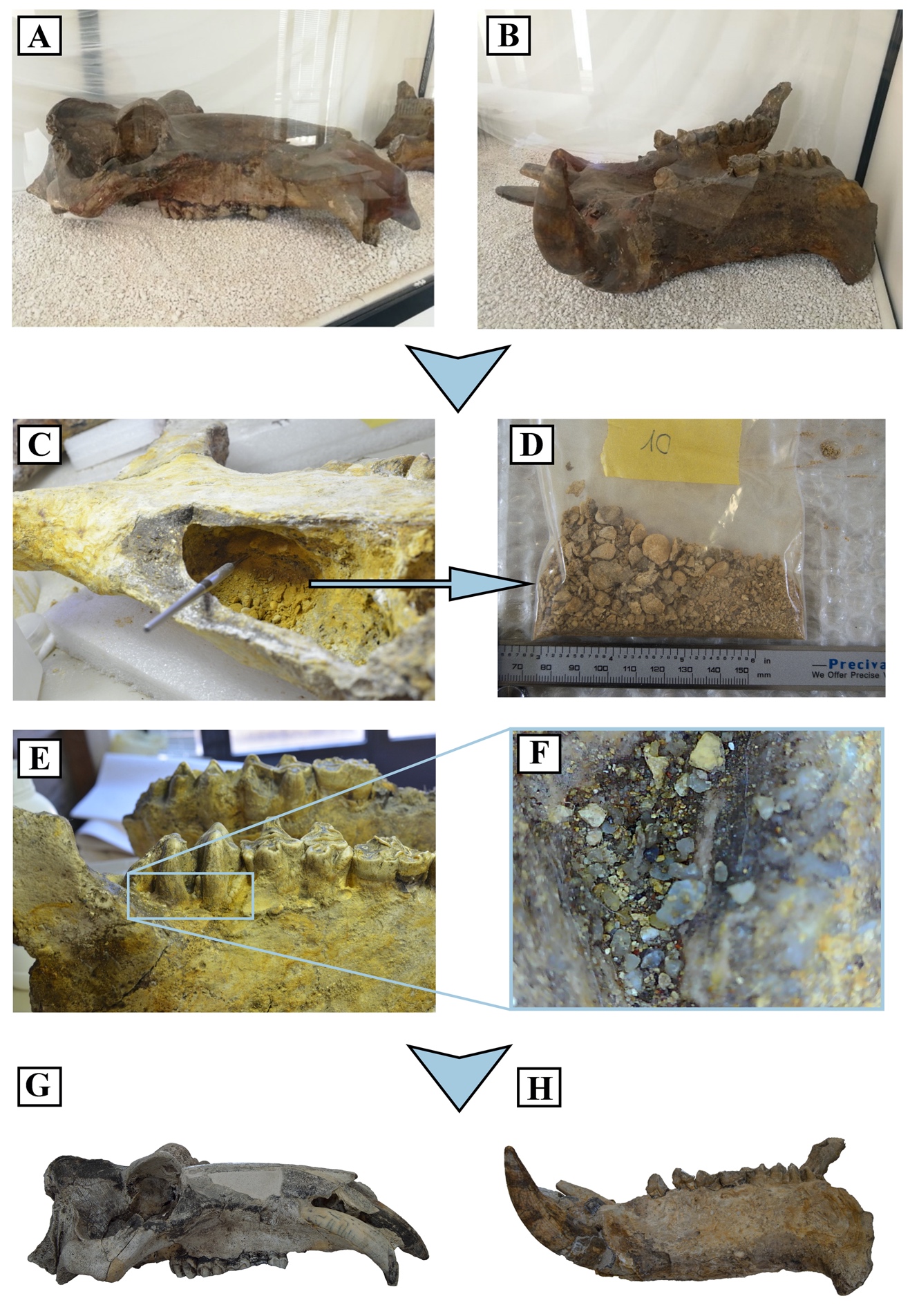


**Fig S1. Hippopotamus skull MPUR/V 149 before the restoration activity (a – cranium; b – mandible); sediments found inside the cavity of the mandible (c) and its sampling for petrographic analyses (d); sediments found around the teeth in the mandible (e – f); the hippopotamus skull MPUR/V 149 after the restoration activity.**

After having removed the integration, sediments were found inside the cavities and encrusted around the basal part of the teeth. These sediments, observed for the first time, were sampled and analyzed in this work.

The last step of the restoration work was to apply the new integration on the skull. In this case, though, a less invasive approach was adopted, avoiding to reconstruct the large missing parts. Concerning the small portion of the bone damaged or broken, a synthethic resin was used. This synthetic resin was colored searching to find a shade similar to the color of the fossil, but slightly different from this so that the reconstructed portion are easily recognizable.

**Sex and ageing**

Completely ossified sutures and permanent teeth (excepted for right DP^4^) allow to refer the SN1139/B specimen to an adult individual. Analysing the molar wear, a further age determination can be proposed following the reference study of Laws (1968). The ontogenetic ages can be obtained considering the age-scoring observed in extant specimens of *H. amphibius*, assuming that fossil hippopotamuses wore their teeth with a similar rate. According to Laws (1968), the MPUR/V 149 specimen can be referred to XII-XIII age classes, which corresponds approximately between 22 and 24 years old.

The extant *H. amphibius* displays size sexual dimorphism, where males are generally larger than females, (Mazza, 1995; Eltringham, 1999; Mazza & Bertini, 2013; Mazza & Ventra, 2011; Shannon et al., 2021). As recently discussed by Shannon et al. (2021), size sexual dimorphism in extant common hippopotamuses is quite reduced, with males slightly heavier (5%), longer (2%) and taller (7%) than females. Clear separation between sexes was found only in the jaw and canines (males are 44% and 81% larger than females respectively; Shannon et al., 2021). Sex differences in canines were generally linked to sexual selection, with largest teeth observed in males (Laws, 1968; Mazza, 1995; Mazza & Ventra, 2011; Shannon et al., 2021).

Considering this, the extant sample of *H. amphibius* has been used to better define the gender of the fossil specimen MPUR/V 149. Unfortunately, the sex of the extant specimens included in our dataset is unknown, therefore we plot the antero-posterior diameter vs. the lateral diameter of the canines and evaluate the related position of the SN1139/B to the variation range of the extant *H. amphibius*.

The values of the C^x^ fell within the range of variation of the extant common hippopotamuses, occupying the right of the plot (Fig. S2). The values of the C_x_ show a similar pattern to that observed in C^x^, but in this case values occupy the central-right position (Fig. S2). In the C_x_, however, two groups would seem apparently distinct, and the MPUR/V 149 specimens fell in the range of variation of the group with largest values. This biometric comparison would support that the specimen MPUR/V 149 belong to a male.


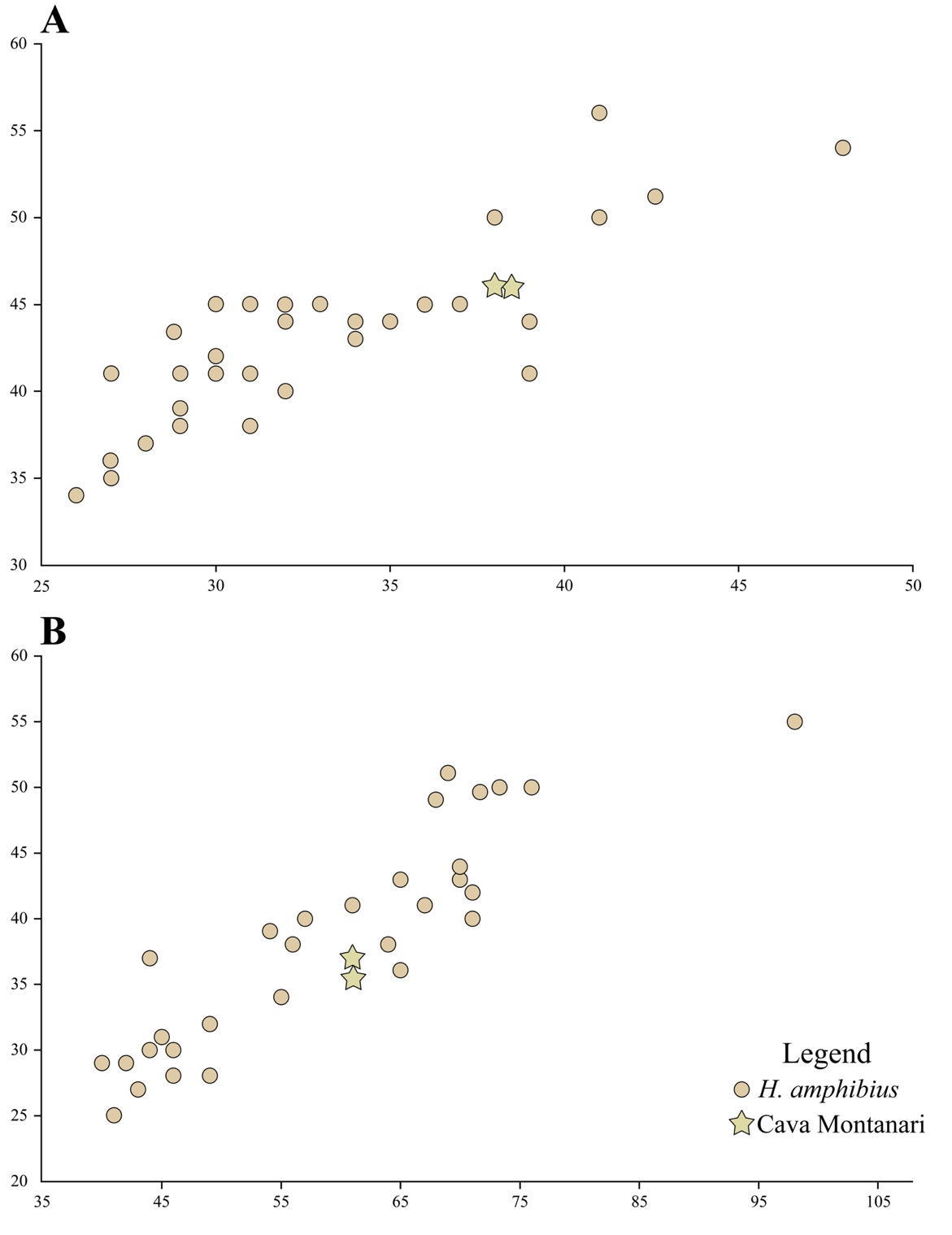


**Fig S2**. **Plot of the antero-posterior diameter vs. the lateral diameter of the upper (a) and lower (b) canines.** Yellow circle - extant specimens of *Hippopotamus amphibius*; Yellow star – Cava Montanari.


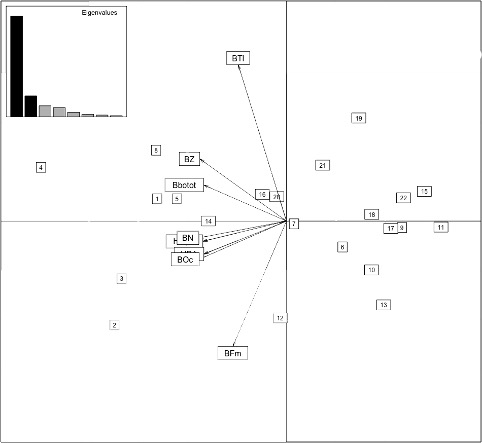


**Fig S3. Contribution of the variables of the Principal Component Analysis (PCA)**


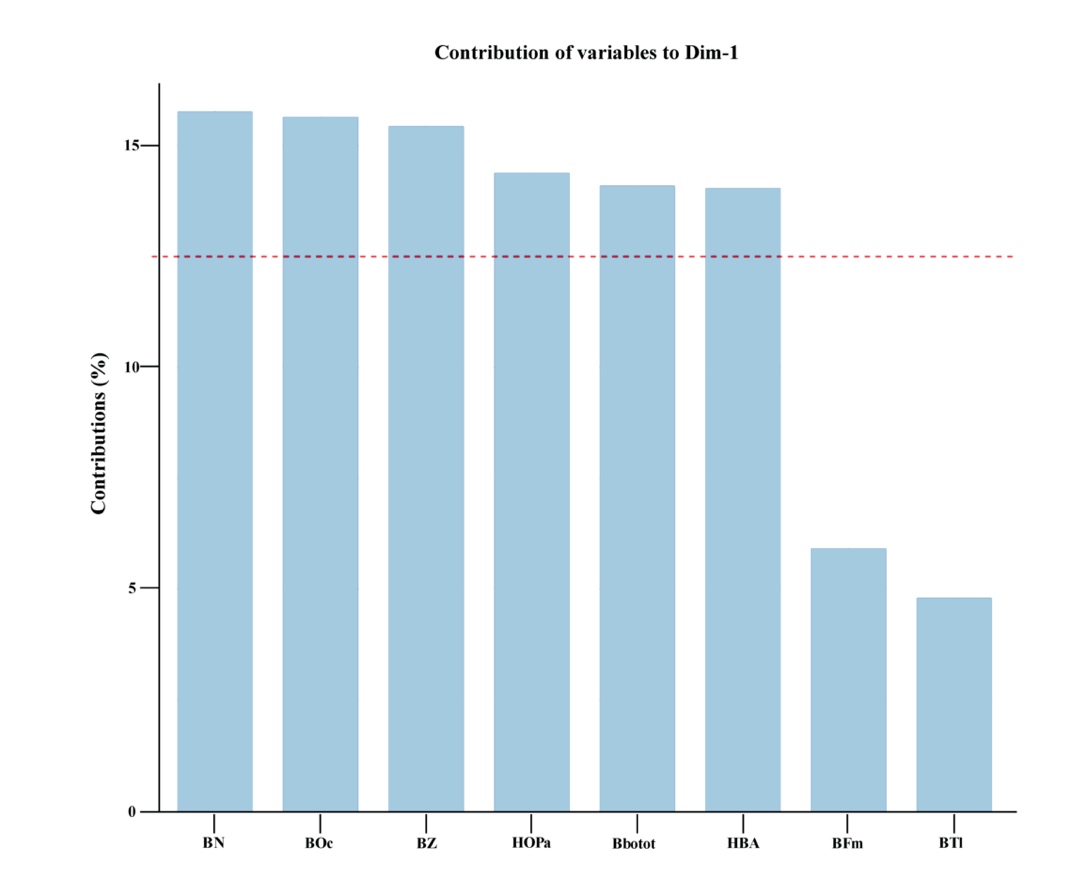


**Fig S4**. **Contribution of variables on first component of the Principal Component Analysis (PCA)**

**
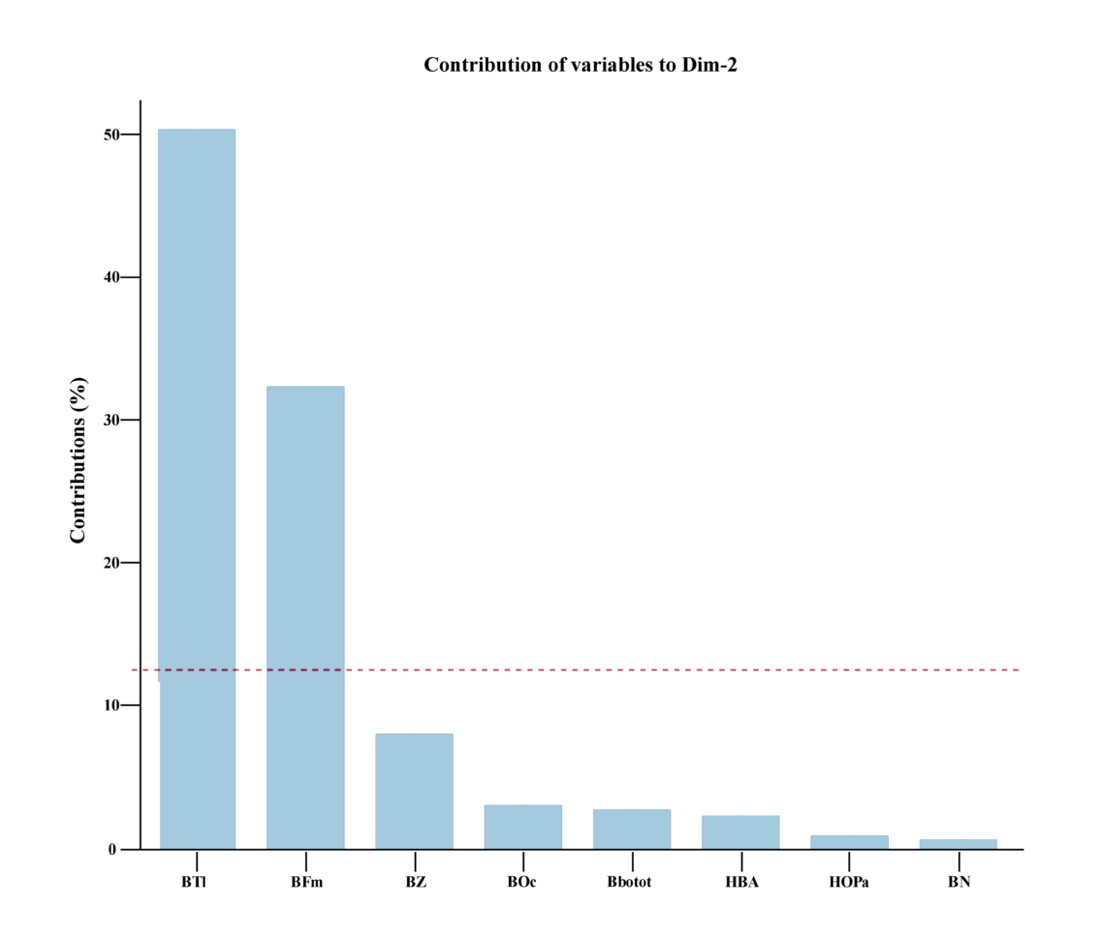
**

**Fig S5**. **Contribution of variables on second component of the Principal Component Analysis (PCA)**


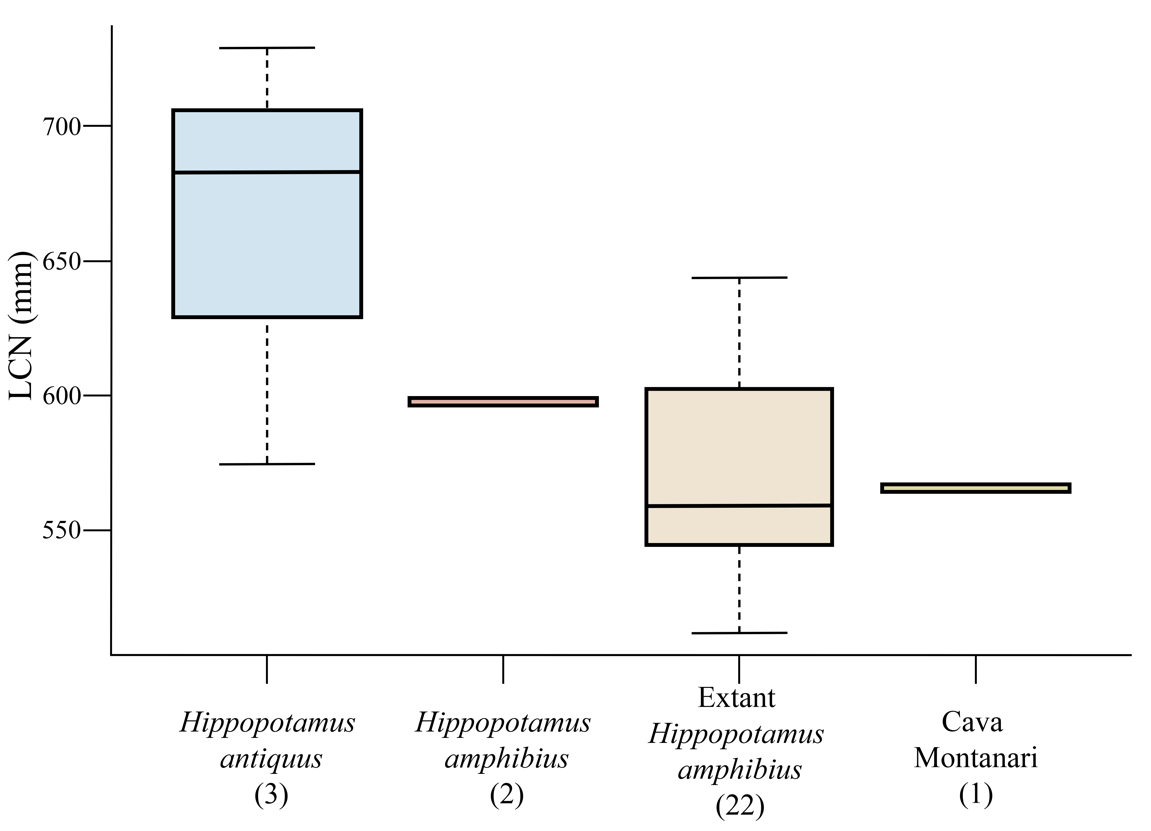


**Fig S6**. **Boxplot of the canine alveolus-nuchal crest length (LCN) of fossil and extant hippopotamuses**.

**
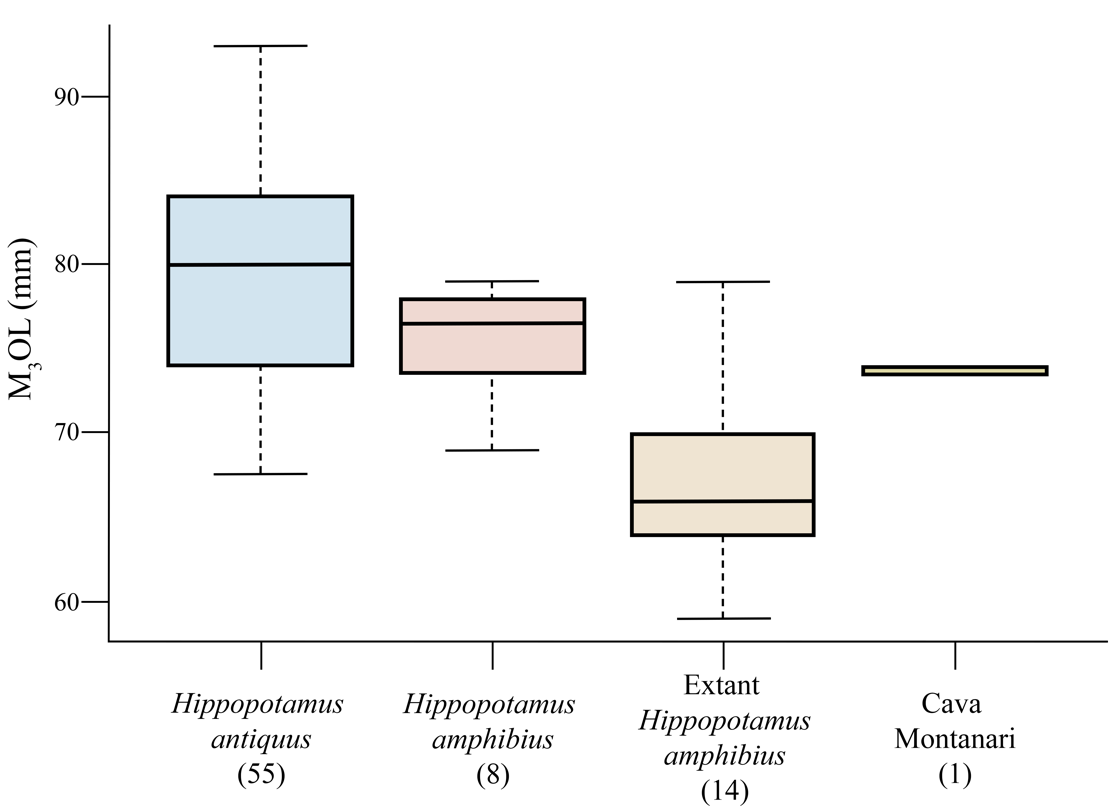
**

**Fig S7**. **Boxplot of the outer length of the lower third molar (M_3_OL) of fossil and extant hippopotamuses.**
